# Hydrogen Recovery from Coke Oven Gas. Comparative Analysis of Technical Alternatives

**DOI:** 10.1021/acs.iecr.1c04668

**Published:** 2022-02-17

**Authors:** Gonzalo Moral, Rafael Ortiz-Imedio, Alfredo Ortiz, Daniel Gorri, Inmaculada Ortiz

**Affiliations:** Department of Chemical & Biomolecular Engineering, University of Cantabria, Av. Los Castros s/n., 39005 Santander, Spain

## Abstract

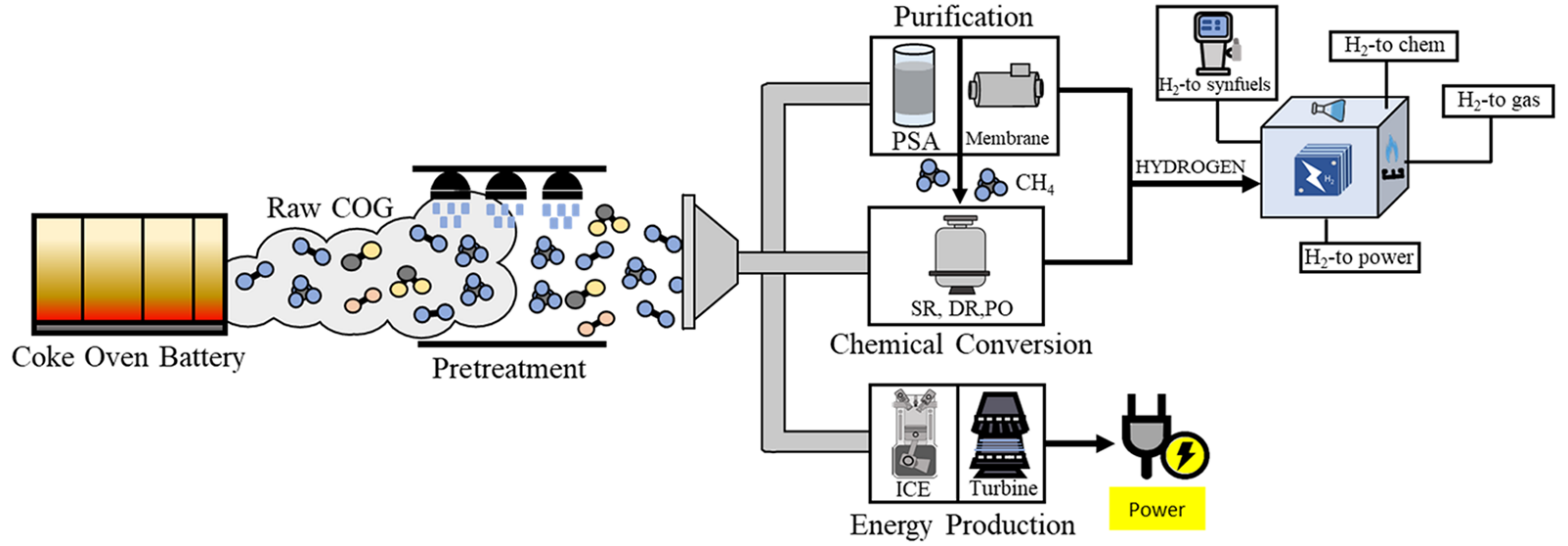

The recovery of energy and valuable compounds from exhaust gases in the iron and steel industry deserves special attention due to the large power consumption and CO_2_ emissions of the sector. In this sense, the hydrogen content of coke oven gas (COG) has positioned it as a promising source toward a hydrogen-based economy which could lead to economic and environmental benefits in the iron and steel industry. COG is presently used for heating purposes in coke batteries or furnaces, while in high production rate periods, surplus COG is burnt in flares and discharged into the atmosphere. Thus, the recovery of the valuable compounds of surplus COG, with a special focus on hydrogen, will increase the efficiency in the iron and steel industry compared to the conventional thermal use of COG. Different routes have been explored for the recovery of hydrogen from COG so far: i) separation/purification processes with pressure swing adsorption or membrane technology, ii) conversion routes that provide additional hydrogen from the chemical transformation of the methane contained in COG, and iii) direct use of COG as fuel for internal combustion engines or gas turbines with the aim of power generation. In this study, the strengths and bottlenecks of the main hydrogen recovery routes from COG are reviewed and discussed.

## 1 Introduction

The exponential growth of the population in the last century together with the associated industrial development has originated a considerable increase in energy demand, that has been mainly supplied from fossil fuels. However, the current carbon-based energy system must cope with the depletion of the global fuel reserves and climate change in the short term, which could lead to an unsustainable situation. Thus, the search for new renewable energy sources and sustainable use of fossil fuels are the main challenges in the energy supply chain roadmap.^1^ With regard to the industrial sector, the iron and steel industry is the largest energy consuming sector, and it accounts for 9% of global carbon dioxide emissions.^2,3^ Steel is made from iron ore as the main iron source, oxygen, and other minerals that occur in nature. Nevertheless, since iron ore contains iron oxide, their sinter (agglomerate of iron oxide fines and other minerals) is previously reduced to iron by the removal of the oxygen content. Coke has been traditionally used as a fuel and reducing agent in blast furnaces, where hot air is injected into the coke, lime, and sinter. Coke is obtained by burning coal in the absence of oxygen at high temperatures in the coke oven batteries. As a result, a solid fraction (coke) and gas fraction (coke oven gas) are obtained. The molten iron from blast furnace is transported to the oxygen furnace, where oxygen is used to decrease the carbon content from 4% to <0.5%.^4^ To overcome the high energy consumption, the iron and steel industry has improved its process efficiency, reducing by 61% the energy required to produce a ton of steel in 2020 compared to 1960.^5^ This context together with the rising price of fossil fuels demands alternatives focused on reducing the energy demand and heat losses and the recovery of valuable compounds contained in waste streams.^6^ In this sense, the waste heat and value compound composition of the exhaust gases such as blast furnace gas (BFG), COG, and Linz-Donawitz converter gas (LDG) could potentially fulfill up to 30% of the energy demand of the iron and steel industry by using them as fuel.^6,7^

Furthermore, COG stands out among waste gas streams due to its high content of valuable compounds (Table 1).

**Table 1:** Composition and Energy Content of Raw and Clean COG^a^

gas composition	units	raw COG^b^	clean COG
H_2_	vol. (%)	39–65	55–60
CH_4_	vol. (%)	20–42	23–27
CO	vol. (%)	4–7	5–8
CO_2_	vol. (%)	1–3	1–2
N_2_	vol. (%)		3–6
C_x_H_y_	vol. (%)	2.0–8.5	1.5–2.3
BTX	g Nm^–3^	20–30	
H_2_S	g Nm^–3^	4–12	≤3.2 × 10^–5^
NH_3_	g Nm^–3^	6–8	
heating value	MJ m^–3^	16–20	

a The information in this table was adapted from refs (8) (with permission of Elsevier) and (9).

b Dry basis. Raw COG contains water vapor (up to 30%) which is removed as the condensate at the pretreatment stage.^9,10^

Approximately 50 Nm^3^ of COG is generated per ton of steel giving 93000 million Nm^3^ of COG produced in 2020.^11,12^ Commonly, there are two ways to cope with coke oven gas. On the one hand, raw COG can be directly used for heating purposes in coke oven batteries or blast furnaces. On the other hand, COG can be cleaned and further processed to obtain valuable products by separation or conversion techniques.^9^ Hence, promoting COG energy recovery pathways is a step forward toward sustainability in the iron and steel industry. Among the valuable compounds, the outlining high content of hydrogen positions COG as a promising source of clean energy. Hydrogen is a feedstock not only in the production of chemicals or refining processes in large scale applications but also in healthcare, food, or pharmaceutical small scale applications. However, the versatility and potential as a fuel source free of greenhouse gas emissions have given rise to a new segment of the market in power generation and the transport sector, where hydrogen acts as an energy vector. So far, the hydrogen demand has been fulfilled by the reforming of fossil fuels, and the obtained product is recognized as “grey hydrogen”. Alternatively, green hydrogen, which is being highly promoted, comes from routes such as water electrolysis using energy from renewable sources. A greenhouse gas emissions-free, hydrogen-based economy places hydrogen as a key element with different purposes: i) to balance the grid when needed using a fuel cell (FC) system (power-to-power), ii) to be blended in the natural gas grid or used as feedstock for synthetic natural gas production (power-to-gas),^13,14^ iii) to be used as fuel in the transport sector (power-to-fuel),^13,15^ or iv) to be employed as a valuable commodity to produce chemical compounds or synthetic fuels (power-to-feedstock).^16,17^ The technological research is being supported by the development of hydrogen policies (30 countries have released hydrogen roadmaps in 2021) in many regions such as Asia, Europe, or Canada.^18−20^ The total investment in hydrogen spending will exceed $300 billion through 2030, and as a result, the hydrogen economy will continue its expansion with a 5.7% growth forecasted for the period 2021–2030.^15^ The future development of hydrogen relies on the reduction of the production costs. In this sense, the rapid global scale-up could drop the electrolyzer system costs from $1120 kW^–1^ in 2020 to $230 kW^–1^ in 2030. Moreover, the cost of renewable energy is falling year-over-year (13% and 9% in solar and wind power, respectively) driven by the infrastructure and equipment development. This context suggests that green hydrogen could be produced for $0.7–1.6 kg H_2_^–1^ before 2050 being competitive with natural gas and fossil fuels.^21,22^ Thus, supplementary sources of hydrogen such as industrial waste streams can contribute to meet the demand after the appropriate recovery process is applied. In this sense, coke oven gas which is presently used as additional fuel in coke ovens or even burnt off in flares is an up-and-coming source of hydrogen. This review discusses the state of the art in hydrogen recovery from COG streams and its further use.

## 2 Hydrogen Recovery from Coke Oven Gas

### 2.1 Pretreatment of Coke Oven Gas

**Figure 1:**
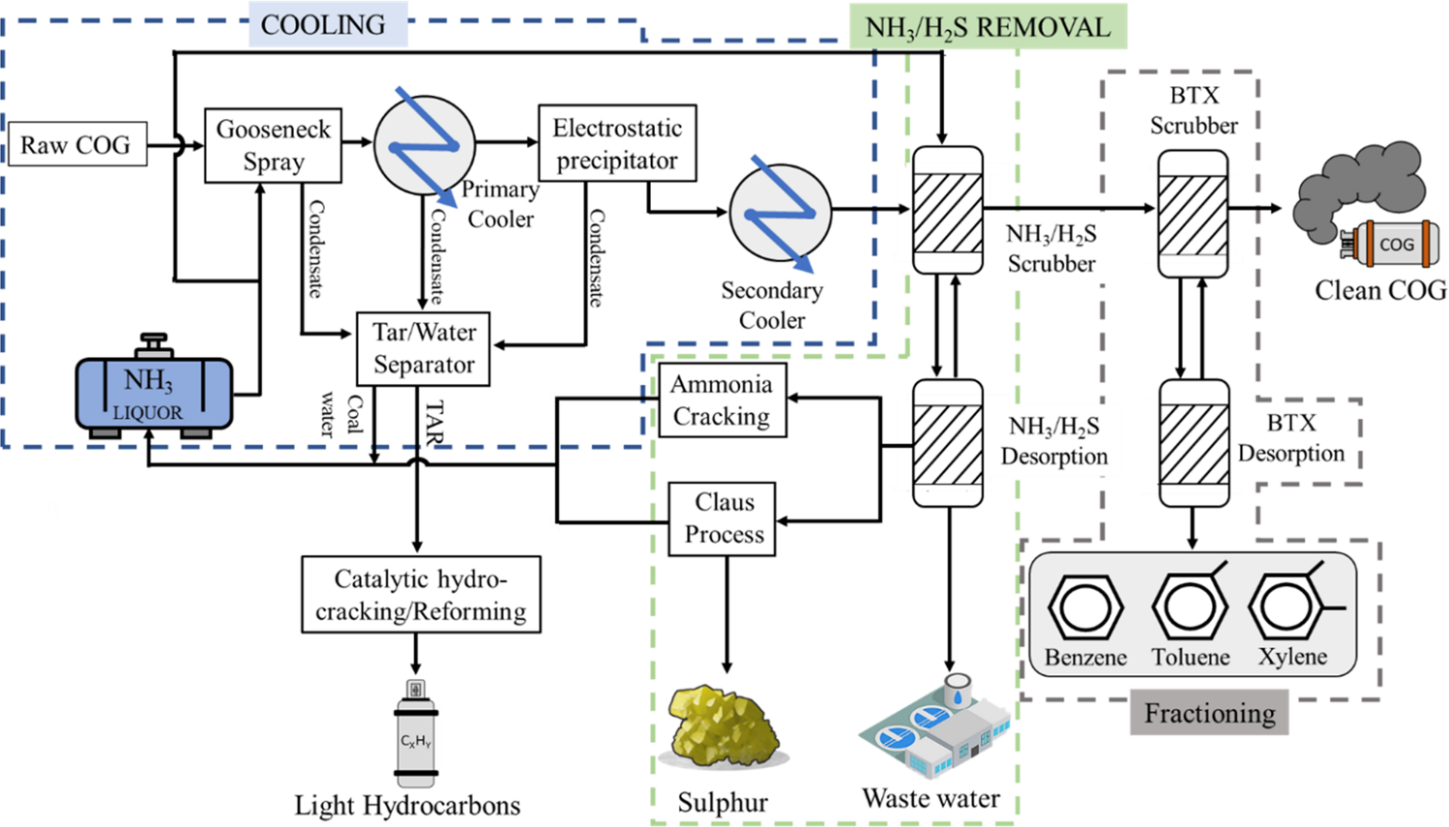
Schematic diagram of the COG pretreatment process, including the potential uses of minor components (adapted from Razzaq et al.^8^ with permission from Elsevier and Remus et al.^9^). The three main stages of COG pretreatment are limited by the dashed lines.

Raw coke oven gas coming out from coke oven batteries contains some minor compounds such as ammonia, tar (semisolid mixture of condensable aromatic hydrocarbons), or hydrogen sulfide, which must be eliminated to prevent fouling and corrosion in pipelines and equipment (see Table 1). Figure 1 shows an illustration of the pretreatment stages (limited by the dashed lines) with the aim of conditioning COG for further recovery.^8,9^

COG is cleaned by the following pretreatment stages:

- Cooling: Raw coke oven gas (1000 °C) is preliminarily cooled by spraying an ammonia solution in gooseneck equipment. Then, gases are further cooled to a temperature of 28–30 °C in direct or indirect coolers, and the fine tar droplets are removed in an electrostatic precipitator. While indirect coolers are shell-and-tube heat exchangers, direct cooling is performed by direct contact with countercurrent streams of ammonia in cooling towers. Subsequently, COG is carried out to washing stages by means of exhausters (suction fans). Since exhausters cause compression of the gas, secondary cooling is necessary in view of attaining the processing conditions for the NH_3_/H_2_S removal stage. Furthermore, tar/water separation of the condensate streams from cooling stages is carried out in a decanter. Finally, tar, which is commonly treated as residue, could be treated by catalytic cracking or reforming reactions to obtain polycyclic aromatic hydrocarbons or hydrogen, while the aqueous solution called “coal water” is fed to the ammonia liquor tank.^23^ Nevertheless, the feasibility of tar recovery is determined after the economic analysis considering that only 25–45 kg of tar can be obtained from each ton of coke, which could question the capital investment.
- NH_3_removal and desulfurization: Ammonia removal and desulfurization stages are carried out by well-known commercial processes. Ammonia can be removed as ammonium sulfate by spraying dilute sulfuric acid solution to the gas or as ammonia solution by water scrubbing. Hydrogen sulfide can be captured by liquid absorption or oxidized by wet or dry oxidative processes to sulfur.^24−26^ Then, the captured H_2_S from absorption could be later transformed to sulfuric acid or sulfur by the CLAUS process.^27^ Although dry oxidation has been historically used, the development of liquid absorption and wet oxidation has neglected this technique, because it entails high cost and space requirements. Additionally, the NH_3_/H_2_S scrubbing-stripping (liquid absorption–desorption) circuit is used with the aim of preventing the production of highly contaminated wastewater from wet oxidation of H_2_S and NH_3_; besides, ammonia liquor could be recovered as a supplementary source for cooling stages of the cleaning process. The process sequence has been detailed by Remus et al.^9^ at the Best Available Techniques reference documents (BREFs). Ammonia is removed from COG in the first scrubber with water. Then, the aqueous solution with ammonia from the first scrubber is used in a consecutive unit as a scrubbing liquor to remove H_2_S. Ammonia and hydrogen sulfide are recovered from the scrubber solution in the stripping stage, and they may be further conditioned. Nevertheless, upgrading of ammonia and hydrogen sulfide streams must satisfy economic feasibility since only 3 kg of NH_3_ and 2.5 kg H_2_S are produced per ton of coke.^9^
- Fractioning: the outlet gas from the NH_3_/H_2_S scrubbing-stripping circuit contains light oil. The main constituents are benzene, toluene, and xylene (BTX). Benzene is primarily used in plastics and resins manufacturing, while toluene and xylene can be used in refineries for gasoline blending.^28,29^ The separation may be accomplished by condensation, gas–liquid absorption, or gas–solid adsorption. Condensation is carried out by a combination of compression and refrigeration steps, which results in high energy consumption and capital investment. Absorption is a mature procedure to recover light oil from COG using creosote or petroleum oil.^30^ Then, BTX are separated from the oil liquor by steam-distillation.

**Figure 2:**
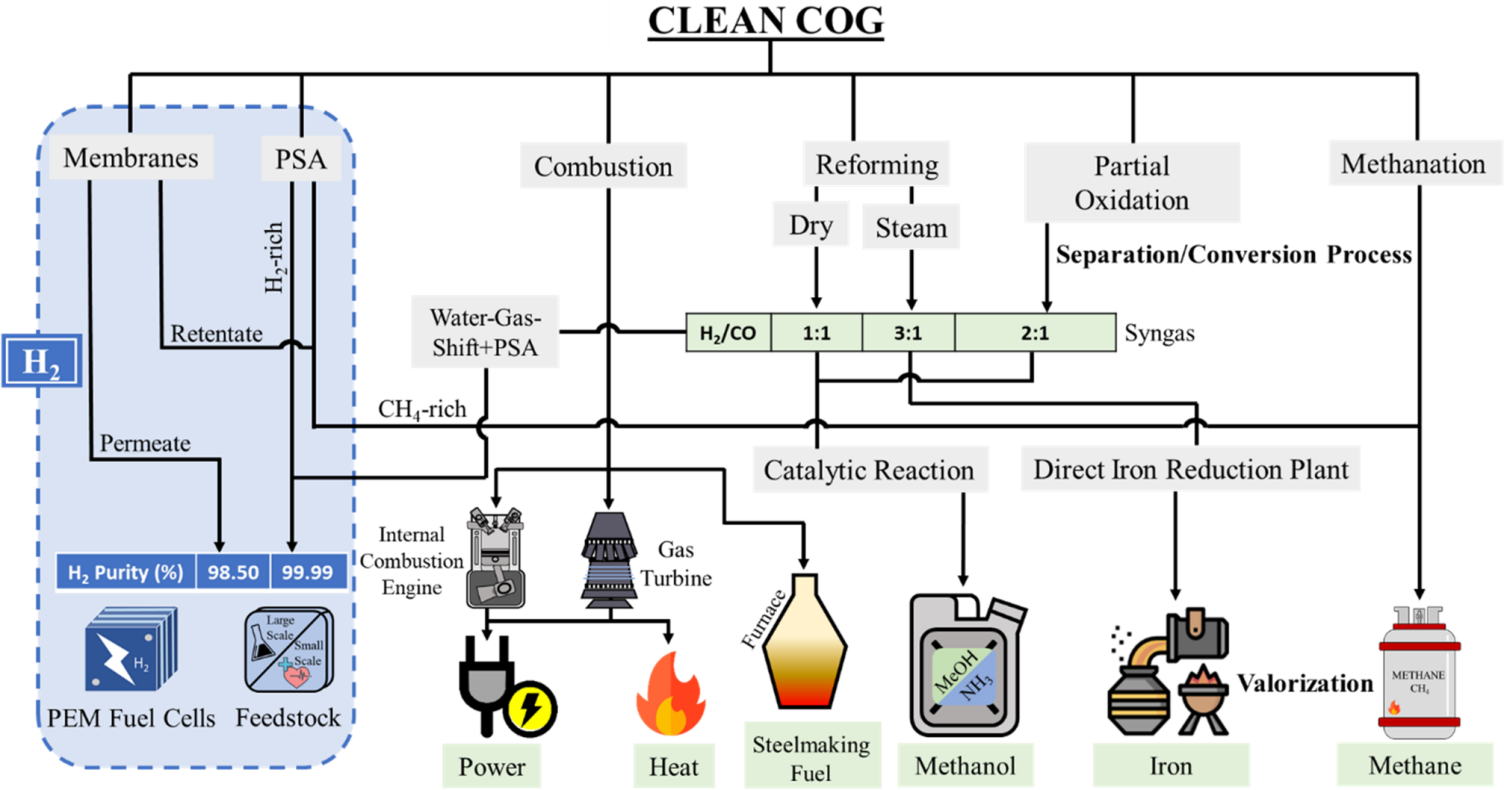
Alternative routes in the recovery of value products from coke oven gas.

Clean COG recovery pathways are summarized in the following section highlighting the production of hydrogen as a valuable product. Figure 2 shows the alternative routes for the recovery of valuable products from COG.

Clean coke oven gas could lead to a wide range of valuable compounds. Hydrogen, which is the most promising product, can be purified by means of separation processes, or it can be obtained from chemical transformations, such as reforming or partial oxidation of the methane fraction of COG. In addition, syngas (H_2_ + CO), which is a feedstock to produce methanol or ammonia, can be obtained in the chemical conversion routes. The H_2_/CO ratio determines the application of the obtained syngas. While higher ratios from steam reforming are suitable for iron reduction in the iron and steel industry or ammonia production (H_2_/CO ≈ 3) by the Haber-Bosch process, lower ratios from partial oxidation or dry reforming fit the requirements for methanol production (H_2_/CO ≈ 2). Furthermore, the hydrogen/methane ratio has positioned COG as a suitable fuel for internal combustion engines or gas turbines for the cogeneration of power and heat to increase the energy efficiency of the manufacturing process. In addition, the upgrading routes can be coupled to increase the recovery of hydrogen from COG in hybrid separation-reaction systems. In this sense, after pretreatment, the clean COG can be subjected to a separation in membrane modules or the PSA unit, obtaining a hydrogen-rich permeate stream, while the methane-rich retentate stream can be subsequently converted into hydrogen by chemical reactions such as reforming or partial oxidation.

### 2.2 Hydrogen Purification

High-purity hydrogen is required for its conversion to electrical energy in fuel cell devices or when it is used as feedstock in manufacturing processes. Commonly, gas separation can be carried out by cryogenic distillation, pressure swing adsorption (PSA), and membrane technology. This review is focused on pressure swing adsorption and membranes because of the large energy consumption of cryogenic distillation, although this technology could be economically feasible to recover hydrogen from purge gas streams in other processes.^31^Table 2 shows the comparison of hydrogen purification techniques.

**Table 2:** Comparison of Hydrogen Purification Techniques^a^

		units	membranes	PSA	distillation
feed requirements		H_2_ vol %	>25	>40	>10
product purity			90–98 (polymeric)/>99.9 (Pd)	>99.9	90–98
operating conditions	temperature	°C	0–100	RT	–183
	feed pressure	bar	20–160	10–40	5–75
hydrogen recovery		%	85–95	50–92	90–99
productivity		Nm^3^ h^–1^	<60,000	30–400,000	10,000–90,000
product pressure		bar	<1/3-feed	feed	feed/low
capital investment			low	medium	high

a The information in this table was adapted from refs (32) and (33) with permission from Taylor & Francis and Elsevier, respectively.

The quality grade required in the produced hydrogen together with the levels of the specific product impurities is critical to the selection of the purification technique. The PSA process is the best choice for high-purity hydrogen production (above 99.9 vol %), whereas polymeric membrane technology is a low-cost alternative to obtain hydrogen of 90–98 vol %, and palladium (Pd) and ceramic membranes are able to reach higher purities (>99.9 vol %). Plant capacity and feed/product pressures should also be considered. Membrane systems are modular, and therefore the costs and production rate are closely related, as capital investment and energy demand are proportional to the number of modules. Besides, PSA benefits from the economy of scale, and it is applicable throughout a full range of capacities and produces hydrogen at feed pressure (10–40 bar), reducing downstream compression costs; this is an advantage when compared to membranes units, where the product is obtained at lower pressures.

#### 2.2.1 Pressure Swing Adsorption (PSA)

**Figure 3:**
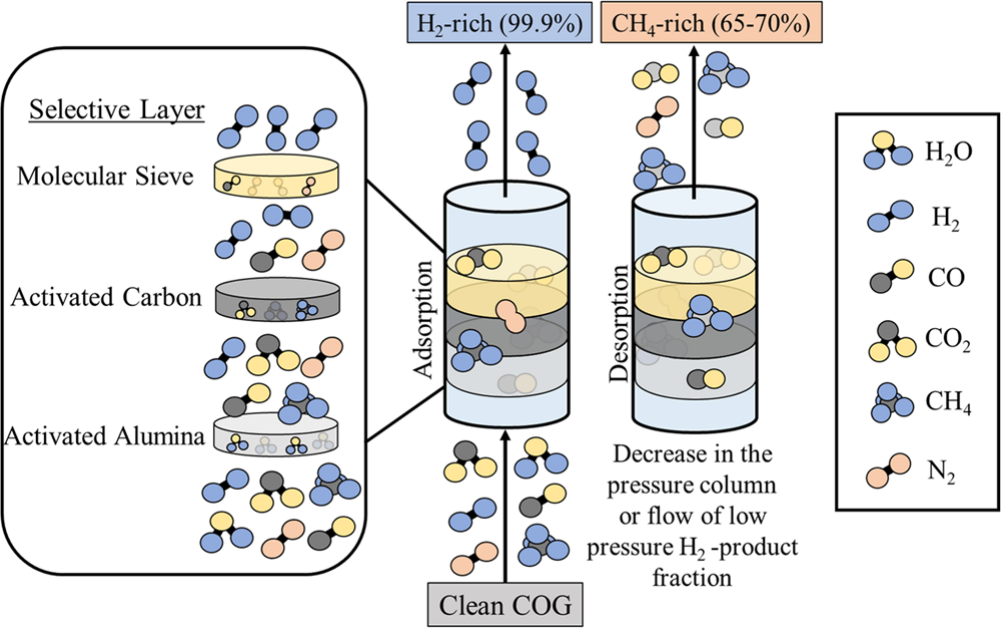
Hydrogen purification from COG by the PSA separation technology.

Pressure swing adsorption is a mature gas separation technology that has been positioned at the forefront for hydrogen purification (85% share of hydrogen purification worldwide) because it allows reaching high purities (>99.99 vol %) and recoveries (70–90%).^34−36^ The process is based on the retention of contaminant molecules in an adsorption bed at high pressures, including some intermediates (methane) and lightly adsorbed components (nitrogen, carbon monoxide). The separation takes place until low adsorbable compounds such as nitrogen or carbon monoxide are not retained in the bed any further and contaminate the product stream (breakthrough time). At that moment, the desorption step starts by means of either decreasing the column pressure or flowing a low pressure fraction of the hydrogen product stream (purge); the adsorbent is regenerated in this step.^34,37^ Thus, it is a cyclic adsorption–desorption operation that gives rise to a H_2_-rich stream (from adsorption) and a CH_4_-rich stream (from desorption). Commonly, the adsorption bed is made of different selective layers. Molecular sieves such as zeolites are used to remove nitrogen and carbon monoxide, which are the most concerning contaminants.^38^ Nevertheless, alumina (AA) and activated carbon (AC) layers must be placed before the molecular sieve to remove water vapor, methane, and carbon dioxide since their strong interaction with zeolites leads to high energy consumption in the desorption stage.^39,40^Figure 3 shows a schematic representation of the PSA separation technology.

The main operating variables in the PSA technology are the adsorption pressure, the purge (referred to the regeneration stage)-to-feed ratio (P/F), and the cycle time. Increasing pressure and P/F increases hydrogen purity since the adsorption mechanism is promoted but at the expense of lower hydrogen recovery and higher energy costs. Moreover, the productivity can be increased by short operation cycles. Since PSA is carried out by adsorption-regeneration cycles, commercial processes are designed with more than four columns to ensure continuous hydrogen production during the regeneration step. Table 3 summarizes the performance data of well-known commercial PSA technologies for hydrogen purification.

**Table 3:** Hydrogen Purification from Commercial PSA Processes^a^

process	licensor	adsorbent	no. of columns	feed^b^	H_2_ purity (%)	H_2_ recovery (%)	capacity (Nm^3^ h^-1^)
Polybed	UOP Honeywell	AC+zeolite 5A	10	SMROG at 21 bar	99.999	86	1000–120000
LOFIN	Toyo Engineering	silica gel/AC	4	ROG at 28 bar	99.6	86.3	5000–200000
Gemini	Air Products	AC/zeolite 5A	9	SMROG at 18 bar	99.99	87	1000–400000

a The information in this table was adapted from ref (35).

b Steam reforming off gas (SMROG), refinery off gas (ROG).

Moreover, the PSA purification process has been registered as the standard technology in several patents for the hydrogen production route from COG.^41−44^ Commonly, high purity hydrogen (99.9% H_2_) and a methane-rich stream are obtained. Chen et al.^41^ have patented a combined PSA-steam reforming process to increase the hydrogen recovery from COG. The hydrogen extracted from COG by PSA together with the hydrogen obtained by the steam reforming reaction of the methane-rich stream from PSA accounts for a recovery of 40,110 Nm^3^ h^–1^ of H_2_ from 50,000 Nm^3^ h^–1^ of COG. The production of high purity hydrogen by PSA still needs to address operational drawbacks such as i) high energy consumption, ii) removal of low absorbable contaminants such as N_2_ and CO which are present in COG, and iii) improving productivity.

One approach to reduce energy consumption involves the use of vacuum in the regeneration stage (VPSA). Since a blower operates at lower pressure ratios than an air compressor of PSA, it is a more energy efficient device. Furthermore, additional equipment such as dryers or filters is not necessary in VPSA, which reduces the capital investment.^45,46^ Golmakani et al.^47^ compared the separation performance and energy consumption of conventional regeneration of PSA (decrease of the column pressure) with alternative regeneration modes such as temperature increase (TSA) and vacuum (VPSA). The experiments were simulated and optimized for a multicomponent feed stream: 75 vol % H_2_, 18 vol % CO_2_, 3.2 vol % CH_4_, 0.7 vol % CO, and 3.1 vol % of N_2_ at 22 bar. Although the three alternatives were designed to obtain 99.995 vol % H_2_, the higher productivity of VPSA (140 mol H_2_ kg_ads_^–1^ day^–1^) compared to PSA (130 mol H_2_ kg_ads_^–1^ day^–1^) together with the lower energy consumption of VPSA (0.94 MJ kg H_2produc_^–1^) compared to TSA (45.44 MJ kg H_2produc_^–1^) positioned VPSA as the best alternative for the regeneration method overcoming the high energy consumption of conventional PSA. Regarding low adsorbable contaminants, the key point is the selection of the molecular sieve layer. In this sense, zeolite 5A and CaX are widely reported in the literature for hydrogen purification from COG. Delgado et al.^48^ have developed the simulation of hydrogen purification from COG in a four-bed PSA process. Zeolite 5A and CaX were selected as adsorption materials achieving almost all fuel cell purity requirements (99.7 vol %) and high recoveries (>70%) at feed pressure of 3 bar in both adsorbent layers.^48^ On the other hand, Ahn et al.^49^ obtained 99.99 vol % H_2_ with activated carbon and zeolite 5A as the molecular sieve layer, working at 10 atm as feed pressure. The analysis of the adsorption curves of a synthetic gas mixture of COG in the AC/zeolite 5A dual layer was reported by Jee et al.^50^ The results confirmed that N_2_ is the less adsorbable compound of COG, which results in the shortest breakthrough time (300 s) at 10 atm of feed pressure. Although the number of works that report experimental results with synthetic gas mixtures with similar composition to COG is steadily growing, alternative approaches for the recovery of H_2_ from binary mixtures to increase the separation performance achieved by PSA are also being considered in the open literature.

The effect of vacuum regeneration and short cycle times was studied in H_2_/CO_2_ binary mixtures by Lopes et al.^51^ Their results showed that a 1-min reduction in the cycle time can increase hydrogen production from 100 to 600 mol-H_2_ kg_adsorb_^–1^ day^–1^. Since the average cycle time in PSA operation is 10–30 min, the reduction of the cycle time could increase the productivity, and the separation could be carried out in smaller columns.

The influence of the P/F ratio was analyzed by Yang et.,^52^ working with H_2_/CO and H_2_/CH_4_ binary mixtures (70/30 vol %) in a two-bed process using zeolite 5A as adsorbent. The results showed that an increase in the P/F ratio results in higher regeneration yield of the bed that ultimately leads to an increase in hydrogen purity. Then, the P/F ratio was optimized by Li et al.^53^ working with a multicomponent hydrogen stream (72.9 vol % H_2_, 3.6 vol % CH_4_, 4.5 vol % CO) and using a dual layer (AC/zeolite 5A) adsorbent. It was found that the P/F ratio should not overpass 0.1 to prevent a significant decrease in the recovery percentage. Regarding new adsorbents for N_2_ and CO impurities, attention has been paid to transition metals with the aim to increase the adsorption capacity of CO. The interaction between transition metals and carbon monoxide by means of reversible complexation reaction results in higher CO adsorption capacity.^54−56^ This solution was studied in multicomponent mixtures (74.36 vol % H_2_, 19.18 vol % CO_2_, 4.01 vol % CH_4_, and 2.45 vol % of CO) by Relvas et al.^40^ The commercial activated carbon was modified by wet impregnation of CuCl_2_-2H_2_O. The product streams showed high H_2_ purity (99.7 vol %) and low CO impurity (0.17 ppm) with 76.2% of H_2_ recovery. Furthermore, the CO adsorbent capacity was increased from 0.35 to 1.25 mol kg_ads_^–1^. Although PSA is a mature technology which has been widely industrialized, there is still room for improvement. The adsorption capacity to low adsorbable contaminants of the selective layer and reduction of the energy consumption should be further improved to meet fuel cell requirements (99.99 vol %, <0.2 CO ppm, <2 CO_2_ ppm)^57^ and ensure the economic feasibility of the process.

#### 2.2.2 Membranes

For many fluid-phase separations, membranes represent a lower investment cost and lower energy consumption option than alternative and more conventional technologies. Commonly, membrane materials can be classified as polymeric (organic), ceramic, carbon, and metallic (inorganic) membranes, although in recent years there has been growing interest in the development of mixed matrix membranes.^58,59^ Ceramic and carbon membranes are microporous materials, which allow hydrogen purification by the molecular sieve mechanism according to the kinetic diameter of molecules. Mass transfer in polymer membranes is usually described by the solution-diffusion mechanism, which assumes that the molecules are absorbed on the membrane surface, then diffuse, and finally are desorbed in the downstream side of the membrane.^60,61^ Temperature and pressure are the main operating variables in membrane separation, while permeability (related to flux) and selectivity (related to purity) are the main characterization parameters. Since polymers are low-cost materials and provide a high degree of separation, research and development in recent decades has resulted in several commercially available membranes for hydrogen separation and purification.^62^ In general, the studies reported in the literature about membranes to separate hydrogen from mixtures classify the membranes in two categories: i) hydrogen-selective membranes, where hydrogen permeates preferentially through the membrane obtaining a hydrogen-enriched permeate stream, and ii) CO_2_-selective membranes, where impurities such as CO_2_ permeate preferentially through the membrane, obtaining a hydrogen-enriched retentate stream.^61^ In the case of hydrogen recovery from COG, H_2_-selective membranes are preferred, since there are no membranes available that are methane-selective. Tables 4 and 5 summarize the characteristics of commercial hydrogen-selective membranes for gas separation.

**Table 4:** Hydrogen Purification from Commercial Polymeric Membranes^b^

membrane	licensor	material	module^a^	H_2_ purity (vol %)	H_2_ recovery (%)	H_2_/CO_2_	H_2_/N_2_	H_2_/CH_4_
PRISM^63^	Air Products	polysulfone	H.F	85–90	80	2.5	56–80	80
ALaS^64^	Air Liquide	polyimide-polyamide	H.F	99.9	96		>200	>200
GENERON^65^	Generon	tetrabromo-polycarbonate	H.F	90–99.9	>90	3.5	90	120
SEPURAN^66^	Evonik	polyimide	H.F		>90			
Polysep^67^	Honeywell	celullose acetate	S.W	>98	95	2.4	72–80	60–81
UBE^68^	Ube Industries	polyimide	H.F			3.8	88–200	100–200

a Hollow fiber (H.F), spiral wound (S.W).

b H_2_ content in feed > 55 vol %.

**Table 5:** Hydrogen Purification from Commercial Metallic Pd Membranes

licensor	material	H_2_ purity (vol %)	flux (Nm^3^ h^-1^)
H2site^69^	Pd	98–99.99	50
Tokyo Gas^70^	Pd-Y(Gd)-Ag/SS	99.9	40
CRI/Criterion^71^	Pd	>99	40–70 Nm^3^ h^–1^ m^–2^ bar^–0.5^
Hysep-ECN^71^	Pd	99.5–99.995	3.6
SINTEF^72^	Pd-Ag	>98	15 Nm^3^ h^–1^ m^–2^

Membrane technology can be also found in patented processes for hydrogen production from COG.^73−75^ As it has been explained in the subsection dealing with the PSA technology, the methane-rich (65 vol % CH_4_) stream may be converted to hydrogen by gas reforming or partial oxidation to increase the hydrogen recovery, or it can be used as supplementary fuel in the plant. Among membrane materials, palladium membranes are selected to obtain high purity H_2_ (99.99 vol %) in separation or hybrid reaction-separation systems. Hydrogen permeation in Pd membranes comprises the adsorption on Pd active sites, the split of the molecule in two protons, the diffusion through the membrane, and recombination on the other side.^76^ Although Pd membranes deliver high separation factors (H_2_/CO_2_: 3147, H_2_/N_2_: 2718), their performance is limited by embrittlement phenomena at low temperature and pressures and poisoning of the membrane when it makes contact with H_2_S, CO, and other compounds.^77^ In this sense, Pd is alloyed with other metals such as silver, copper, or gold to ensure stability in long time operations.^78,79^ The influence of the alloying element on the performance was discussed by Al-Mufachi et al.^80^ While Pd-Y membranes deliver the highest H_2_ permeability (3.7–5) × 10^–8^ mol m^–1^ s^–1^ Pa^–0.5^ at 350 °C), Pd-Cu exhibits higher mechanical stability and sulfur deactivation resistance. Moreover, the development of membranes with a higher flow of hydrogen is necessary to increase the cost-effectiveness of separation. Thus, research is focused on the production of membranes with a thin layer of palladium on a porous support. Itoh et al.^81^ prepared a thin film of Pd (2–4 μm) and H_2_/N_2_ selectivity of 5000 supported on alumina tubes. The preparation of Pd membranes by physical vapor deposition was studied by Pereira et al.^82^ A thin film (1 μm) of Pd supported on alumina with H_2_ permeance of 0.21 × 10^–6^ mol m^–2^ s^–1^ Pa^–1^, at 300 °C, was observed. Finally, Goldbach et al.^83^ obtained a Pd-Au layer supported on a ceramic composite membrane by an electroless plating method. The thin dense layer (3–5 μm) permits high H_2_ permeability (1.3 × 10^–6^ mol m^–2^ s^–1^ Pa^–0.5^ at 300 °C) and H_2_/N_2_ selectivity (1100) at 500 °C.^83^

**Figure 4:**
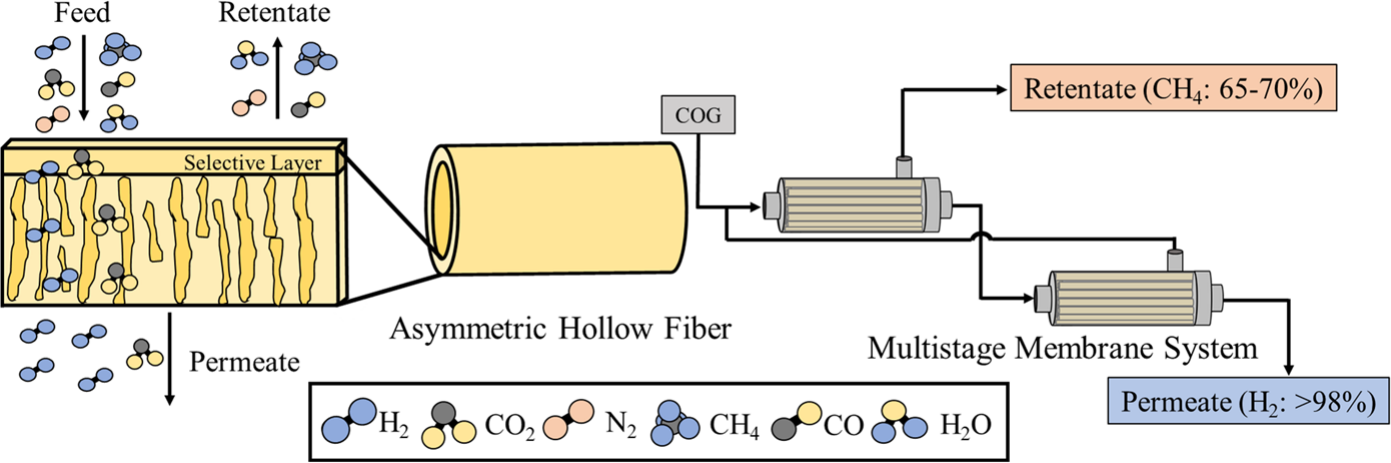
Hydrogen purification from COG by membrane separation technology.

Despite the commercial and patented membrane technology providing hydrogen of high purity degree and recovery, one single-stage membrane process can very rarely meet both requirements, except in the case of using high-cost palladium membranes. For that reason, multiple membrane stages, i.e., membrane cascades, are routinely employed, as shown in Figure 4.^84^ Numerous studies have been published in the literature on the synthesis and optimization of gas permeation membrane networks, describing various possible configurations for the membrane cascades.^85^ However, membrane systems consisting of a series of two or three stages represent the optimum configurations from the techno-economic point of view.^86,87^ The selection of the cascade configuration is determined by the feed gas composition, pressure ratio, product purity, and product recovery. Among these, membrane selectivity is the most influential factor.^84^ Moreover, research has focused on developing tailor-made and low-cost polymeric membranes with higher performance. Nevertheless, to the best of our knowledge, few studies have been reported in the open literature on hydrogen recovery from COG by membranes. Yañez et al.^88^ evaluated the performance of three commercial membranes for the recovery of hydrogen from synthetic feed gas whose composition was similar to the industrial purge streams (including COG, methanol, and ammonia purge streams). The experiments were carried out at 5.5 bar of transmembrane pressure difference in a temperature range of 25–45 °C for polyetherimide (PEI), polyethersulfone (PES), and polybenzimidazole (PBI) membranes. The permeability increased with temperature as expected since the components’s diffusion is enhanced by temperature. Hydrogen permeabilities in the range of 5–9.4 Barrer were observed for PEI and PES, while PBI delivered lower permeability (≈1 Barrer). Moreover, H_2_/CO_2_ was found to be the key and bottleneck separation mixture since hydrogen selectivity is 10 times lower compared to H_2_/N_2_ and H_2_/CH_4_ mixtures. The experimental results with gas mixtures showed that PEI (4–4.9) delivers double selectivity values than the PES membrane (1.7), and a slight increase in selectivity with temperature was observed. The same trend was found by Ansaloni et al.^89,90^ using a cross-linking polyimide(PI)-silsesquioxane(POSS) dense layer supported by γ-alumina hollow fibers. These authors reported that selectivity significantly decreased (by 50%) in gas mixtures with respect to the ideal selectivity evaluated from the permeation experiments carried out with pure gases. The following selectivity values were reported for H_2_/N_2_, H_2_/CH_4_, and H_2_/CO_2_, respectively, in gas mixtures (H_2_ permeability, 150 Barrer): 5–8, 5–10, 2–2.5.

Although the recovery of the components of coke oven gas separation requires further research, the development of high-performance membranes for hydrogen purification is a topic of enormous interest to the scientific community. In this sense, polymer blending, pyrolysis (thermal annealing) of polymer precursors to obtain carbon membranes, and doping with inorganic fillers stand out to address hydrogen purification for fuel cell applications.^91^ Acharya et al.^92^ analyzed the behavior of the performance of polysulfone(PSF)/polycarbonate(PC) membranes for the separation of H_2_/CO_2_ mixtures. An increase in H_2_ permeability (from 13.5 Barrer to 25 Barrer at 50 wt % of PSF/PC) was observed, while the selectivity is inversely proportional (from 2.52 to 1.17 at 50 wt % of PSF/PC) to the concentration of PC compared to pristine PSF. Moreover, Matrimid polyimide membranes have been widely used in the synthesis of polymer blends for hydrogen purification.^93−96^ The influence of pyrolysis of Matrimid-blends to obtain carbon membranes was reported by Hosseini et al.^94^ Results showed that carbon PBI/Matrimid membranes surpass the Robeson upper bound for hydrogen separation from nitrogen, carbon dioxide, and methane.

Carbon molecular sieve (CMS) membranes are produced by pyrolysis of polymeric precursors. The degradation of the polymeric chains leads to the formation of porous structures (<0.6 nm) which increase the selectivity through the molecular sieve mechanism. The selection of the polymeric precursor and the operating variables of the thermal treatment determines the membrane structure and the separation performance. Lei et al.^97^ studied the separation performance of carbon hollow fibers of cellulose precursor. The membranes were fabricated by the dry-wet spinning process and carbonized at 550 to 850 °C. Results exhibited 83.9 H_2_/CO_2_ selectivity with 148.2 GPU of H_2_ permeance as the best overall performance (*T*_pyrolysis_: 850 °C). Nevertheless, the selectivity increases 4 times by the increase in the pyrolysis temperature, while permeance decreased 3.6 times. Xu et al.^98^ prepared CMS by the pyrolysis of phenolphthalein-based cardo poly(arylene ether ketone) (PEK-C) at 700 °C. The membranes showed high H_2_ permeability (5260 Barrer) and selectivity (H_2_/N_2_: 142, H_2_/CH_4_: 311, H_2_/CO: 75). In addition to traditional polymer precursors, graphene-based membranes have gained attention in the recent years. Since defect-free graphene is impermeable to all gases, single layer studies focus on the development of different techniques (UV-oxidative etching or ion beam milling) to create subnanometer pores that can act as gas transport channels. On the other hand, multilayer graphene membranes deliver high performance and simpler manufacturing processes to cope with the bottlenecks of single-layer membranes.^99^ Li et al.^100^ developed a thin graphene oxide multilayer (9 nm) supported on alumina by vacuum filtration. The membranes were tested with binary hydrogen mixtures (50/50 vol % H_2_/CO_2_ and 50/50 vol % H_2_/N_2_) and exhibited high H_2_/CO_2_ (3400) and H_2_/N_2_ (1000) selectivity and flux (H_2_ permeance 300 GPU) at 20 °C. Moreover, multilayer configuration allows the manufacturing of hollow fiber membranes facilitating industrial applications. In this sense, the synthesis of graphene membranes (320 nm selective layer) supported on alumina hollow fiber was studied by Huang et al.^101^ The separation performance of the membrane (H_2_ permeance: 400 GPU, H_2_/CO_2_: 15) positioned the results beyond the Robeson’s upper bound. Despite multilayer configuration showing high performance, graphene membranes show a decrease in selectivity in humidity atmospheres. Since graphene is a hydrophilic material, the water vapor trend to condense on the surface or inside the pores leads to a significant reduction of the separation performance.^102^ In this sense, an interesting approach was reported by Huang et al.^103^ Positively charged nanodiamonds were incorporated into the graphene oxide layers. The results showed that the graphene/nanodiamond membrane retains up to 90% of H_2_ selectivity in an aggressive humidity test.

Inorganic fillers such as zeolites and metal organic frameworks (MOFs) have received great attention in the last decades to improve the hydrogen selectivity delivered by pristine polymers. Mixed matrix membranes (MMMs) combine the molecular sieve mechanism due to the filler microstructure together with an increase in the polymer free volume, which results in an increase in hydrogen selectivity and permeability avoiding the pyrolysis treatment. The effectiveness of MMMs relies on the pore size of the filler and the compatibility with the polymer. In this sense, zeolites are microporous crystalline aluminosilicates which have been used in a wide range of applications.^104^ Regarding hydrogen purification, zeolites with intermediate pore size between H_2_ (2.89 Å) and CO_2_ (3.3 Å) kinetic diameter are highly desirable. Among the studies reported in the literature, it was observed that the use of zeolites 4A and 3A as fillers provides the higher increase in selectivity.^105−109^ Ahmad et al.^108^ showed an increase in H_2_/N_2_ selectivity of 37% when 25 wt % of zeolite 4A was added to polyvinyl acetate. Khan et al.^109^ found an increase by 2.3 times in the H_2_/CO_2_ selectivity with 40 wt % of zeolite 3A incorporated into polysulfone acrylate membranes. ZIFs (Zeolitic Imidazole Frameworks) a subset of MOFs, which easily interact with polymers and facilitate hydrogen permeation flux, have been also investigated as fillers.^110^ Addition of ZIF-8 to different polymers provides higher H_2_/N_2_, H_2_/CH_4_, and H_2_/CO selectivity, while the selectivity of binary H_2_/CO_2_ mixtures slightly increases compared to the pristine Matrimid polymer, because the pore size of the ZIF-8 (3.4 Å) is placed between H_2_, CO_2_, and bulk compounds: N_2_ (3.64 Å), CO (3.76 Å), CH_4_ (3.8 Å).^111−113^ Besides, Diestel et al.^111^ reported an increase in H_2_/CO_2_ selectivity with ZIF-90 in the Matrimid polymer matrix. Overall, according to the reported literature, the use of inorganic fillers could result in the increase of both the selectivity and the permeability. Although promising, mixed matrix membranes must face challenges and further development to assess the technology scale-up and industrialization. The filler to polymer ratio requires further investigation and optimization. Ratios up to 35 wt % are recommended because higher ratios can lead to weaker structures and lower selectivity performance due to the excessive increase in the free volume which results in higher permeabilities of bulk compounds such as N_2_, CH_4_, and CO. Moreover, the scale-up of membrane technology is based on hollow fiber configuration and multistage membrane systems. In this sense, further investigation on the manufacturing of hollow fiber mixed matrix membranes together with design and optimization of multistage membrane systems for hydrogen recovery from COG is required. In addition, the modularity of membrane technology has resulted in hybrid configurations with PSA with the aim of reducing the costs of producing high-purity hydrogen. In this sense, the selection of the configuration (PSA-Membrane, Membrane-PSA, Membrane-PSA-Membrane) and the optimization of the operating parameters are the main challenges that must be addressed. Li et al.^114^ compared the performance of PSA-Mem and Mem-PSA with conventional PSA for the purification of hydrogen from coal gasification syngas (62.57% H_2_, 31.61% CO_2_, 4.33% N_2_, 1.12 CO, and 0.37% CH_4_). Results showed an increase of 40% in hydrogen recovery of the PSA unit in hybrid configurations in the production of high purity H_2_ (99.98%). Although hybrid systems allow an increase in the recovery of the process, the selection of the configuration must meet the product specifications and financial profitability. The technical and economic analysis of hybrid separation processes was carried out by Lin et al.^115^ The study evaluates the separation of the H_2_-N_2_ mixture from the decomposition of ammonia with PSA, membrane, and hybrid processes. Results showed that hybrid configurations with more stages such as Mem-PSA-Mem increase the energy consumption. On the other hand, since high-purity hydrogen is obtained in the PSA unit, configurations in which hybrid PSA is placed before the membrane unit is placed before membrane units are recommended, from an energy efficiency point of view. The tail gas is from PSA, which is fed to the membrane unit, where the permeate stream is fed back into the PSA unit. This design decreases the stream flowrate through the membrane module, which reduces the energy consumption in the compression stage. PSA-Mem delivers the lowest cost ($4.31 kg H_2_^–1^) compared to Membrane-PSA ($4.47 kg H_2_^–1^), conventional PSA ($5.54 kg H_2_^–1^), or Pd membranes ($5.39 kg H_2_^–1^) for the separation of high purity hydrogen (99.97%).

Finally, dense ceramic membranes have become a hot topic as novel membranes for hydrogen purification. The transport mechanism involves the following steps: i) H_2_ adsorbs onto the membrane surface and dissociates into protons and electrons and ii) protons and electrons diffuse to the other side of the membrane, where they recombine to H_2_. Theoretically, the hydrogen selectivity of the mixed proton–electron conducting membranes is 100% as in the case of Pd membranes. Since ceramic membranes are less expensive and have a greater resistance in H_2_S, CO, and CO_2_ atmospheres, they are well-positioned for the purification of hydrogen at high temperatures such as those employed in membrane reactors. Nevertheless, the commercialization of proton–electron conducting membranes is still hampered by insufficient stability in long-term operations, low proton and electron conductivities which lead to lower H_2_ flux, and fundamental knowledge of the membrane performance.^116,117^ Thus, research focuses on the development of membranes containing electron and proton conducting phases, doping of the membranes, and the investigation of novel materials such as La_2_Ce_2_O_7_ oxides. Since dense ceramic membranes are still in their early days, the open literature focuses on the characterization of hydrogen flux by pure gas experiments; thus, studies on hydrogen separation from multicomponent gas mixtures are lacking. A comprehensive review of future trends and the summary of hydrogen flux in dense ceramic membrane can be found in Tao et al.^118^

## 3 Coke Oven Gas Chemical Conversion to Feedstock

### 3.1 Reforming and Partial Oxidation

Pressure swing adsorption and membrane technology are separation methods which also produce a methane-rich byproduct stream which could be burnt as fuel. In this sense, upgrading techniques such as reforming or partial oxidation of COG provide syngas from the reaction of methane. Then, hydrogen is obtained by means of the water-gas-shift (WGS) reaction and downstream purification step such as PSA.^119^ Thus, hydrogen recovery from COG by separation steps is complemented with the hydrogen product obtained from methane conversion. Nevertheless, with the high value of syngas as feedstock in manufacturing processes, the chemical conversion to H_2_ in WGS reactors is not always considered an option. Moreover, all the proposed methods are based on the catalytic conversion in fixed bed or fluidized reactors which requires a previous cleaning process with the aim of preventing poisonous effects on the catalyst.

#### 3.1.1 Steam and Dry Reforming

**Figure 5:**
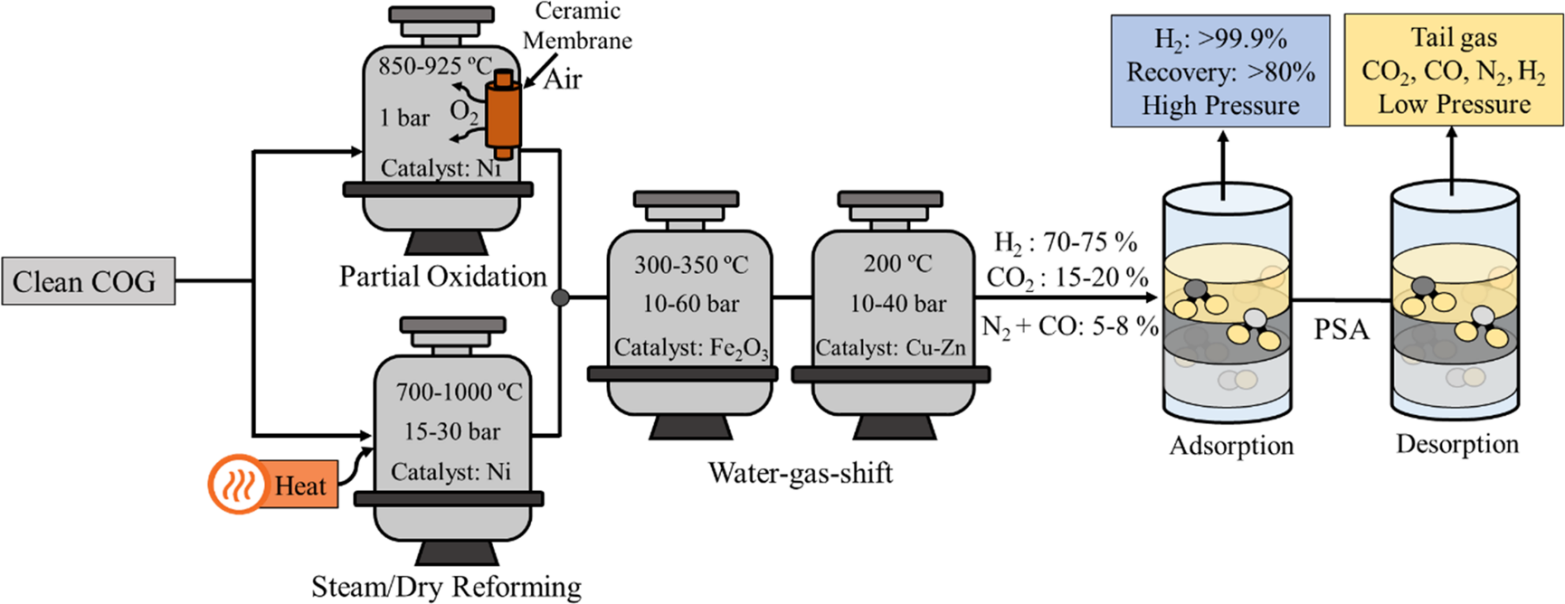
Hydrogen production by reaction routes of COG.

Steam reforming (SR) is the main process for syngas and hydrogen production (Figure 5). The process consists of a heterogeneous catalyzed reaction of the methane fraction of COG with high temperature steam (700–1000 °C, 15–30 bar) to obtain syngas with the H_2_/CO ratio of ideally 3/1 (reaction 1).^120^ Among the catalysts, Ni stands out from the noble metals (Ru, Rh, Pd, Ir, or Pt) due to its lower price. Nevertheless, Ni delivers the lower activity (≈94% CH_4_ conversion) and deactivation resistance to carbon deposition or sulfur poisonous compounds.^121−123^ Moreover, the selection of the catalyst morphology depends on the operating conditions. Large particles with thick walls such as six-hole cylinders offer high resistance to temperature and mechanical stress.^27^ After steam reforming, an additional amount of hydrogen can be obtained from syngas by the water-gas-shift reaction (reaction 2).

Commonly, the WGS reaction takes place in two reactors. First, the high amount of carbon monoxide is converted until reaching equilibrium in a high-temperature reactor at 300–350 °C with iron oxide-based catalysts. Then, the outlet stream is cooled down to 200 °C and further converted (90–99% CO conversion) using a copper-zinc catalyst supported on alumina or silica.^122,124^
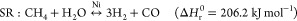
1
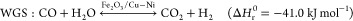
2

**Figure 6:**
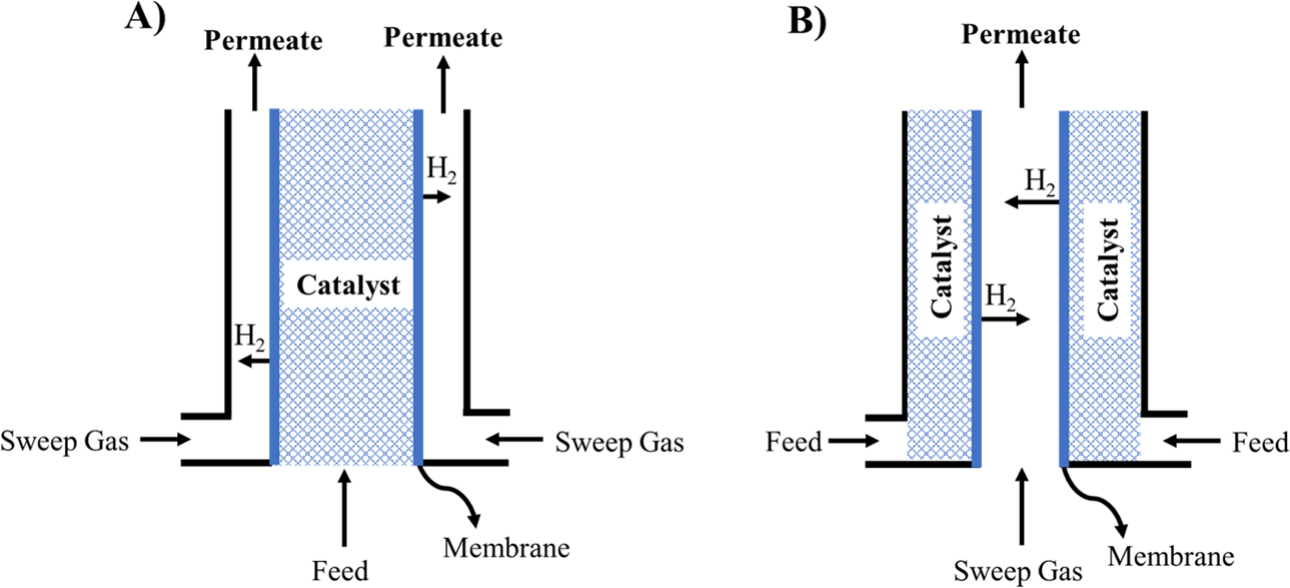
Membrane reactor configuration in cocurrent flow. Catalyst in the inner tube (A) and catalyst in the outer shell (B).

Temperature, pressure, and the steam to carbon ratio (S/C) are the main operating variables of the process. The production of high purity hydrogen from COG requires advanced separation-reaction systems (sorption-enhanced (SE) or membrane assisted (MA) steam reforming reactors), since the initial content of hydrogen and carbon monoxide in COG induces unfavorable reactions such as the reverse-water-gas-shift (RWGS). The main goal of separation-reaction systems is the increase in the reactant conversion by the removal of reaction products from the reactor that shifts the equilibrium to higher conversions (up to 35% higher than conventional reactors).^125^ In this sense, while hydrogen is selectively recovered by membranes, sorption-enhanced systems rely on the capture of the carbon dioxide which is produced in the WGS reaction on an adsorption bed. In addition, membrane reactors allow operating at lower reaction temperatures, reducing the capital and operational costs by the lower energy consumption and materials costs. Moreover, this introduces the development of new strategies of heat integration for the off-gases of the processes.^126,127^ Membrane reactor configuration generally presents shell and tube configuration in cocurrent flow. The catalyst may be placed at the inner of the tube or in the annulus, while permeate flows in the remaining section.^125,128^ The schematic representation of the configuration is shown in Figure 6.

The selection of the operating variables of the MA reactors must meet the reaction and separation requirements. In this sense, temperature ranges between 400 and 600 °C, which enhance the reactants conversion and hydrogen permeation, reducing the energy consumption compared to conventional SR reactors. Regarding pressure, reaction and separation show competitive effects. While the conversion of the reactants is unfavored by an increase of the pressure, the driving force for gas transport is enhanced. Thus, mild pressures (1–10 bar) are commonly used in MA reactors.^125^ The shift from conventional reforming to new separation-reaction systems can be either observed in patented processes or in the open literature for hydrogen production from COG. Regarding the registered technology, metallic membrane reactors have been patented in the past decade.^129,130^ On the other hand, studies of steam reforming of COG are scarce to the best of our knowledge since the process is still at its early stages. The performance of a separation-reaction system for the production of hydrogen from COG was evaluated by Chen et al.^131,132^ High purity hydrogen (>99.9 vol %) was obtained in a MA-SE-SR process from COG at 560 °C with an S/C ratio of 4. Calcined dolomite was used as the adsorbent for carbon dioxide capture, while a palladium membrane selectively separated hydrogen (2.14·10^–2^–3.34·10^–2^ mol m^–2^ s^–1^) from the reaction medium. Moreover, the analysis of the influence of the carbon dioxide capture on the steam reforming of COG was studied by Wu et al.^133^ An increase from 78 to 95.8 vol % in H_2_ purity from SR to SE-SR of COG was observed. Moreover, as it has been mentioned in the Membranes section, proton conducting membranes are well positioned for membrane reactor applications. Although studies in the open literature are scarce, these novel membranes could provide higher energy efficiencies than conventional MA reactors as reported by Malerød-Fjeld et al.^134^ They produce high purity hydrogen with full methane conversion (99%) in a protonic membrane reformer (PMR) at 800 °C. Thus, an almost pure carbon dioxide membrane is also obtained. Furthermore, the modeling of the process showed that PMR requires 1/3 electricity and 2/3 natural gas compared to a traditional MA reactor.

Dry reforming (DR), which consists of the reaction of methane and carbon dioxide (reaction 3), can be promoted during steam reforming.
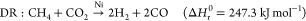
3

Dry reforming has the advantage of using both greenhouse gases for syngas production with a low H_2_/CO ratio (1/1). Nevertheless, the reaction requires high temperatures (>800 °C) because of its endothermic character. Thus, the open literature focuses on the enhancement of the catalyst activity. Li et al.^135^ reported the increased activity of monometallic catalysts and the resistance to carbon deposition by the Ni-Co bimetallic catalyst with 70.36% and 86.46% conversion of methane and carbon dioxide, respectively, at 700 °C. The influence of the catalyst in the reaction was observed by Angeli et al.^136^ Their results showed that higher temperatures (1100 °C) are required to carry out the dry reforming of BFG and COG in the absence of a catalyst (78.5% of CO_2_ conversion and 95% CH_4_ conversion). Combined steam and dry reforming reactions were studied by Kim et al.^137^Lower carbon dioxide (25–34%) and methane conversion (81–87%) were observed compared to dry reforming, while a H_2_/CO ratio slightly higher than 3 was obtained. Although the reforming reaction requires separation-reaction systems or downstream hydrogen purification to meet fuel cell requirements, this alternative is well positioned to increase the recovery of hydrogen from COG. Moreover, a reforming reactor can be also placed after the separation process by membranes or PSA to further transform the methane-rich stream to hydrogen.

Regarding syngas production, the ratio H_2_/CO is determined by the selection of the reforming process. While higher ratios obtained from steam reforming (>3) are suitable when syngas is used as a reducing agent in iron production, lower ratios obtained from dry reforming (≈2) are required in methanol production which could be obtained by partial oxidation (PO) of COG.

#### 3.1.2 Partial Oxidation

The partial oxidation (PO) of methane unlike steam and dry reforming is an exothermic process that does not require an external source of energy (equation 4).^120^ Commonly, Ni-based catalysts are used to promote the reaction rate and selectivity.
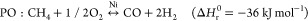
4

According to the stoichiometry of reaction 4, ideally a 2:1 H_2_/CO ratio is obtained by the partial oxidation reaction; this fulfills the requirements for methanol production. Then, hydrogen can be also obtained by means of the water-gas-shift reaction followed by a purification step. The main challenge in partial oxidation is the supply of high purity oxygen. Conventionally, pure oxygen has been produced from the cryogenic distillation of air at the expense of high energy consumption. In this sense, attention has been paid to oxygen-selective ceramic membranes, which integrate oxygen separation and PO reaction in a single stage; this integrated step provides significant reduction in energy demand and capital investment. This approach is found in the open literature of hydrogen production by partial oxidation of COG.^138−144^ Furthermore, the oxygen permeable reactor has been patented for the partial oxidation of COG.^145^ Nb-perovskite-based ceramic membranes (BaCo_0.7_Fe_0.2_M_0.1_O_3-δ_, recognized as “BCFM”) where “M” used to be a transition metal such as Nb, Ta, or Zr and “δ” is the concentration of oxygen vacancies in the structure are widely studied. The performance of the membrane reaction system was studied by Yang et al.^143^ and Zhang et al.^141^ The methane conversion and oxygen flux ranges from 90 to 95% and 15–17 mL cm^–2^ min^–1^, respectively, at 875 °C. Moreover, Cheng et al.^139^ studied the influence of the transition metal on the stability of the perovskite membrane. In spite of the slight increase in permeation flux with Zr, it was found that BCFZ membranes have lower structural stability in the CO_2_ atmosphere. The partial oxidation technology has been also patented for the production of syngas from COG.^146,147^ Thus, according to the state-of-the-art literature, research should be focused on the development of oxygen-selective ceramic membranes with higher stability and permeation flux to offer a more advantageous chemical transformation route for the recovery of hydrogen from COG.

### 3.2 Methanation

Methanation consists of the conversion of CO_2_ (reaction 6) and CO (reaction 5) to CH_4_:^148^
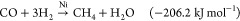
5
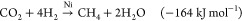
6

**Figure 7:**
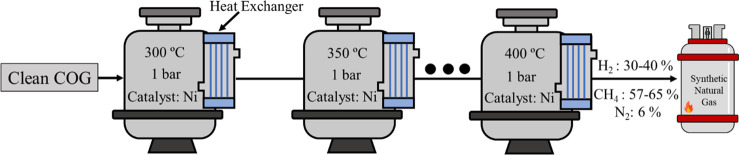
Production of synthetic methane from COG.

Thus, COG can be used to provide the reagents in the methanation reaction. Methanation has recently gained attention in power-to-gas applications in which hydrogen excess is used for synthetic methane production from CO_2_ toward the reduction of fossil fuels consumption and carbon dioxide emissions.^149^ Conventionally, methanation is a catalytic reaction which is carried out in adiabatic reactors. Although methanation was discovered at the end of the 19th century, it still remains as a new alternative in the recovery routes of COG. In this sense, a methanation process has been patented with in-series adiabatic reactors.^150,151^ Nevertheless, the literature review shows that there are two main obstacles to be overcome in methanation: i) catalyst performance and ii) temperature control. Since it is a catalytic reaction, many studies focused on increasing the catalyst activity and the deactivation resistance. In this sense, bifunctional Ni-based catalysts have been widely reported. Lu et al.^152^ observed the enhancement of the activity and stability of the Ni catalyst with zirconia (Ni-Zr) to reach 100% and 80% conversion of CO and CO_2_, respectively, at 450 °C. Moreover, Ni-Ce catalysts were tested by Quin et al.^153^ The results showed complete conversion of carbon monoxide and carbon dioxide at 260 °C. On the other hand, the exothermic character of the reaction together with the high concentration of reactants results in a significant increase in the temperature of the reactor. Thus, heat exchangers should be coupled to the adiabatic reactors to control the temperature of the process.^154^ The comparison between conventional adiabatic reactors and nonadiabatic reactors was studied by Quin et al.^148^ Nonadiabatic reactors delivered higher production ratios (20%) and lower costs (14%) due to the reduction of the necessary equipment. Figure 7 shows the illustration of the methanation process of COG.

## 4 Coke Oven Gas Combustion to Energy

**Figure 8:**
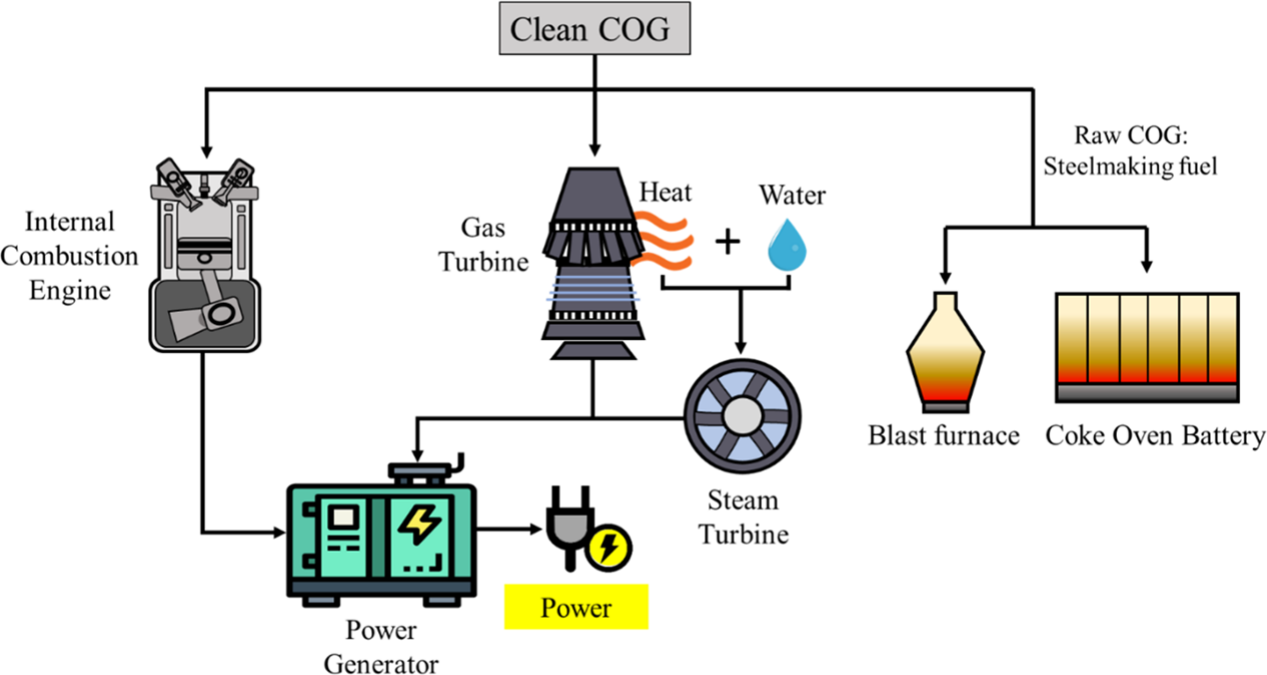
COG energy recovery in ICE and turbines.

Among nonstandard gaseous fuels, COG has a high heating value (16–20 MJ m^–3^), which allows the gas to be burnt at a normal temperature, while the blast furnace gas, with a one-tenth heating value of the natural gas (3–5 MJ m^–3^), requires higher temperatures.^155,156^ In this sense, raw COG, which is sometimes flared off during periods of lower demand, has been commonly fed to furnaces and coke oven batteries accomplishing a low cost reuse standard. However, hydrogen and methane concentration in COG has given rise to unprecedented recovery routes such as feedstock in cogeneration or internal combustion engines with the aim of power and heat the coke at the iron and steel industry, reducing the energy demand (Figure 8).^157^ Regarding cogeneration, modeling and simulation of cogeneration studies are focused on the optimization of exhaust gases allocation in the plant.^157,158^ The optimization of the utilization of COG and LDG in the iron and steel plant was studied by García et al.^157^ Mixed integer linear programming (MILP) was used as a tool for the allocation of the streams. Results showed an increase of 16.9% of the benefits by the MILP model since it allows the optimization of the performance of the cogeneration plant, while human decision-making is only focused on the reduction of natural gas consumption. On the other hand, COG can be fueled in two types of internal combustion engine devices: turbines and reciprocating engines. Some modern gas turbines, e.g., GE 6B gas turbine, are fuel flexible and can be fed by liquid or gaseous fuels, such as COG.^159−161^ Gas turbines can burn COG with compressed air, propelling the rotation of the shaft with the combustion gases and producing electricity with a generator connected to the same shaft. To further achieve a higher system efficiency, a combined-cycle gas turbine (CCGT) can be used, in which the exhaust gases can be used to heat water through a heat recovery steam generation (HRSG).^162,163^ The steam produced is then introduced in a steam turbine connected to the same or another generator. Therefore, gas turbines are a very efficient and high-power density technology; however, they are expensive and require a very specialized maintenance. In contrast, reciprocating internal combustion engines (ICEs) are easily scalable to the plant requirements, are cheaper than gas turbines, and require low specialized maintenance. In order to be fueled with gaseous fuels, a preliminary conditioning is required to tackle combustion differences from oil conventional fuels (diesel and gasoline), optimizing the operating conditions. The necessary modifications in ICEs are related to design: i) higher capacity injectors due to the lower density of hydrogen-rich mixtures which results in larger fuel volumes, ii) spark plugs and better cooling systems able to manage higher combustion temperatures, and iii) other minor instrumentation, such as a wideband lambda sensor to operate at leaner mixtures.^164,165^ Two main injection configurations are usually employed. Port-fuel injection, which requires low-pressure injectors, provides a more homogeneous air-fuel mixture and increases the combustion efficiency, but a higher backfire tendency and lower power output due to the less volumetric efficiency are obtained.^166,167^ On the other hand, direct fuel injection into the cylinder increases the power performance because of the higher mass of air induced and richer air-fuel mixtures can be employed without the risk of backfire. Nevertheless, high-pressure injectors are required, and higher thermal NOx should be controlled as higher combustion temperatures are reached.^166^ Studies of ICEs fueled with gaseous fuels have grown exponentially in the last decades. A tradeoff between higher efficiency but lower output power operating at lean air-fuel mixtures has been found in hydrogen internal combustion engines.^168^ In addition, leaner mixtures avoid abnormal combustion and reduce NOx emissions, especially at optimum spark advance.^169^ Spark advance also influences the maximum brake torque, becoming an important factor for the optimization of the operating conditions, as observed by Sopena et al.^165^ In order to increase the power performance while reducing knocking at richer air-fuel mixtures, blends of H_2_ and CH_4_ can be used as gaseous fuels. In this sense, a wider operating range can be employed, limiting the combustion temperature and duration.^170−172^

Thus, cleaned COG, which is mainly composed of hydrogen and methane as shown in Table 1, is a very interesting industrial waste stream to harness its energy content. Different studies of the combustion of COG or similar gas compositions in internal combustion engines are found in the literature. Regarding compression ignition engines, COG and a pilot amount of diesel have been tested and compared with producer gases with different H_2_ percentages and pure H_2_ in a supercharged dual-fuel engine by Roy et al.^173,174^ Higher H_2_ content increased the efficiency but reduced the output power and the emissions as leaner air-fuel mixtures were required to avoid knock, observing an important influence of the air-to-fuel ratio and the timing of the pilot diesel injection.

In the case of spark ignition engines, gas mixtures similar to COG were tested and compared with other synthesis gases with different compositions.^175,176^ Results showed good combustion stability of COG and suitable antiknock properties of CH_4_, CO, and CO_2_.^176^ In addition, knocking was reduced similarly by diluting the fuel mixture by means of EGR or by leaning the air-to-fuel mixture with an excess of air.^175^ Comparing a methanized COG mixture of 55 vol % of H_2_ and 45 vol % natural gas (NG) with NG and a mixture with 30 vol % H_2_ and 70 vol % of NG, higher efficiency and NOx emissions were obtained with the methanized COG mixture but produced lower torque and low emissions of CO and HC.^177^ An availability analysis (maximum useful work that can be produced from a system during the interaction to a state of thermal, mechanical, and chemical equilibrium with its environment) for COG, methane, and a mixture of 80 vol % of H_2_ and 20 vol % CH_4_ was carried out, delivering the highest thermal efficiency and the lowest specific fuel consumption with COG.^178^ Additionally, it was found that the irreversibility could be reduced by increasing the compression ratio and delaying the spark timing. On the other hand, Ortiz-Imedio et al.^179^ compared hydrogen, methane, and a synthetic COG mixture, observing a widening of the air-fuel ratio operation range with COG and obtaining lower specific NOx, hydrocarbon, CO, and CO_2_ emissions. Moreover, a computational fluid dynamics (CFD) simulation showed that intermediate spark advance values of COG reduced the combustion pressure and temperature within the cylinder, decreasing NOx emissions and the wall heat transfer. In this way, COG generated the highest power values compared to CH_4_ and H_2_ at lean air-to-fuel mixtures.^180^

García et al.^181^ analyzed the environmental impact of the energy recovery of waste streams in steel production by means of the life cycle analysis tool. Coke oven gas and Linz-Donawitz converter gas were evaluated as supplementary fuels to natural gas in different scenarios that were defined according to the energy contribution of natural gas and off-gases. The authors reported environmental benefits in human toxicity (evaluation of toxic compounds for the human health), ionizing radiation (damage to human health and ecosystems that are associated with the emissions of radionuclides), fossil and ozone depletion indicators (depletion of natural fossil fuel resources and emissions to air that cause the destruction of the stratospheric ozone layer, respectively), and natural gas savings (120 Nm^3^ MWh^–1^ in 100% of energy production from COG and Linz-Donawitz gas) in all the analyzed scenarios.^182^ Furthermore, it was demonstrated that the higher the energy recovery from waste gases the greater the benefit. In conclusion, the high-energy content of coke oven gas can be harnessed in a controlled way through its combustion in both gas turbines and reciprocating internal combustion engines. A wider operating range of air-to-fuel ratios compared to H_2_ and CH_4_ can be employed, taking advantage of the individual benefits of its main constituents. High thermal efficiency and output power values are obtained, while lower hydrocarbon emissions compared to conventional fuels and lower NOx emissions than pure H_2_ are generated. Therefore, COG as an industrial waste stream is a very interesting alternative for energy production in the iron and steel industry, reducing the energy demand from more polluting fossil fuels.

## 5 Environmental Analysis of the Valorization Routes

Among coke oven gas valorization routes, the production of electricity and heat is positioned as the cheapest alternative. Nevertheless, the sustainability of the valorization routes must be addressed according to economic and environmental aspects. In this sense, the emissions of carbon dioxide are the main bottleneck in the valorization of COG. Since the production of iron and steel is an energy intensive industry, the selection of the upgrading technique should be focused on the reduction of greenhouse gas emissions. A comparison of the environmental performance of the valorization routes of COG was performed by Zhang et al.^183^ The study evaluated the energy consumption and carbon dioxide emissions of the alternatives that have been discussed in previous sections (Table 6).

**Table 6:** Carbon Dioxide Emissions and Energy Consumption of COG Valorization Routes

valorization route	CO_2_ emissions (kg CO_2-eq_ $^-1^)	energy consumption (MJ $^-1^)
conversion to electricity and heat	9.1	136.6
hydrogen purification	7.0	177.8
chemical conversion to feedstock (methanol)	8.6	175.5
conversion to feedstock (methanation)	6.2	184.6

As can be seen in Table 6, the environmental performance of hydrogen purification stands out compared to the cogeneration of heat and electricity, which is currently the most economic option since the low energy consumption. Moreover, the recovery of hydrogen from COG has been compared to alternative hydrogen production routes in recent studies.^184,185^ The global warming potential of hydrogen production from COG is in the range of natural gas reforming (10–13 kg CO_2-eq_ kg H_2_^–1^) and only decreased by water electrolysis with renewable energy sources. Although the recovery of hydrogen from COG must face economic drawbacks, the growth of hydrogen economy together with the environmental performance could position this alternative at the head of valorization techniques of COG in the midterm.

## 6 Conclusions and Future Prospects

Among exhaust gases of the iron and steel industry, COG stands out as a promising hydrogen sustainable source. Although raw COG is used as a supplementary fuel, the high production rates in the iron and steel industry result in surplus COG which is usually burnt off in flares. Thus, COG as a hydrogen source, after the appropriate conditioning, has attracted much attention due to the environmental and economic potential toward sustainability and a hydrogen-based economy. In this sense, two main pathways are distinguished in the recovery of hydrogen from COG: i) separation/purification process and ii) chemical conversion from methane and carbon dioxide contained in COG combined with separation/purification steps. Furthermore, the hydrogen and methane composition in COG positions it as suitable fuel for H_2_-fueled internal combustion engines or gas turbines in stationary applications to supply electricity and heat to the iron and steel plant. Regarding hydrogen recovery, the selection of the alternative route depends on the purity of the hydrogen product, capital investment, and operation costs. According to the literature research, hybrid separation-reaction systems are well positioned to maximize the hydrogen recovery from COG. Since the initial composition of hydrogen in COG unfavored the conversion of methane by shifting the equilibrium of the reaction, membrane technology can be placed prior to the conversion step as the first hydrogen recovery stage. Then, the methane-rich stream can be converted to syngas by reforming or partial oxidation and further processed to hydrogen by the water-gas-shift reaction. Finally, the product stream from the WGS reactor (70–75% H_2_) should be purified by the PSA process to meet fuel cell purity requirements. Thus, hybrid separation-reaction systems allow an increase in the hydrogen production since the initial content in COG is enhanced by the chemical transformation of methane to hydrogen. Nevertheless, separation and chemical transformation routes must overcome operating drawbacks to address the economic feasibility of the process (Table 7). Regarding separation technologies, lower energy consumption from PSA and higher separation performance are required. In this sense, the operation of the regeneration stage under vacuum conditions allows the reduction of the energy consumption and the capital investment. Regarding membrane technology, the selection of the membrane material depends on the operating conditions. While Pd and proton conducting membranes are the best alternative for the recovery of hydrogen at high temperatures such as those employed in membrane reactors, polymeric-based materials deliver high separation performance at lower operation temperatures such as the initial recovery of hydrogen from COG previous to the chemical conversion route. However, polymeric-based membranes are not able to meet the high purity requirements hampered by the separation of hydrogen and carbon dioxide. Thus, the studies focus on the doping (mixed matrix membranes) or conditioning of the membranes (carbon membranes) to increase the separation grade. On the other hand, the increase in the catalyst activity and deactivation resistance is required in the chemical conversion routes to hydrogen to ensure long-term operation and reduction of the energy requirements. Regarding the increase in catalyst activity, bifunctional Ni-based catalysts are widely found in the open literature, while advanced membrane-reaction integrated systems have shown lower energy requirements and capital investment than conventional reaction systems.

**Table 7:** Bottlenecks and Future Prospects of Hydrogen Production Routes from COG

process	technology	bottleneck	R&D trend
hydrogen recovery	PSA	N_2_ and CO low adsorption contaminants	transition metal to enhance CO adsorption
		high energy consumption to reach fuel cell	vacuum regeneration
		tail gas utilization	
chemical conversion to feedstock	membranes	increase of H_2_/CO_2_ selectivity to reach fuel cell purity	Pd membranes
			proton conducting membranes
			carbon membranes
			mixed matrix membranes
		retentate valorization	feed to chemical conversion process for hydrogen or syngas production
	reforming and partial oxidation	H_2_ and CO in COG: unfavored reactions (RWGS)	advanced reaction-separation systems: membrane (Pd and conducting membranes) and sorption enhance reactors
		energy consumption and capital investment	
		catalyst deactivation	Ni-Mx/support (where Mx is metal or metal oxide)
			Mx: increase activity and stability (i.e., Zr, Ru, Rh, Co, Ir)
			support: increase deactivation resistance (i.e., alumina, calcium aluminate, magnesium aluminate)
		oxygen supply in partial oxidation	oxygen-selective ceramic membranes
	methanation	temperature controlling	heat exchanger reactor
		catalyst deactivation	same trend as that in reforming and partial oxidation
conversion to energy	combustion	reduce abnormal combustion and increase the output power of the ICEs	utilization of turbocharger
			optimization of direct injection
			exhaust gas recirculation
		reduce NOx emissions	increase the compression rate

## References

[ref1] International Energy Agency. Net Zero by 2050. https://www.iea.org/reports/net-zero-by-2050 (accessed 2021-10-13).

[ref2] Bermúdez J. M.; Arenillas A.; Luque R.; Menéndez J. A. An Overview of Novel Technologies to Valorise Coke Oven Gas Surplus. Fuel Process. Technol. 2013, 110, 150–159. 10.1016/j.fuproc.2012.12.007.

[ref3] Wang P.; Ryberg M.; Yang Y.; Feng K.; Kara S.; Hauschild M.; Chen W. Q. Efficiency Stagnation in Global Steel Production Urges Joint Supply- and Demand-Side Mitigation Efforts. Nat. Commun. 2021, 12, 2066 10.1038/s41467-021-22245-6. 33824307 PMC8024266

[ref4] AcelorMittal. Making Steel. https://corporate.arcelormittal.com/about/making-steel (accessed 2022-01-18).

[ref5] World Steel Association. Energy Use in the Steel Industry; Brussels, 2021.

[ref6] Wang R. Q.; Jiang L.; Wang Y. D.; Roskilly A. P. Energy Saving Technologies and Mass-Thermal Network Optimization for Decarbonized Iron and Steel Industry: A Review. J. Clean. Prod. 2020, 274, 122997 10.1016/j.jclepro.2020.122997.

[ref7] Zhang Q.; Liu W. C.; Du T.; Cai J. J.; Xu C. B.; Bai X. B. Utilization Secondary Energy in Integrated Iron and Steel Works for Improving Energy Utilization Efficiency. Proc. - 2010 Int. Conf. Digit. Manuf. Autom. ICDMA 2010 2010, 2, 887–889. 10.1109/ICDMA.2010.412.

[ref8] Razzaq R.; Li C.; Zhang S. Coke Oven Gas: Availability, Properties, Purification, and Utilization in China. Fuel 2013, 113, 287–299. 10.1016/j.fuel.2013.05.070.

[ref9] Remus R.; Aguado Monsonet M.; Roudier S.; Delgado Sancho L. Best Available Techniques (BAT) Reference Document for: Iron and Steel Production: Industrial Emissions Directive 2010/75/EU: (Integrated Pollution Prevention and Control); EUR 25521 EN; Publications Office of the European Union: Luxembourg, 2013; 10.2791/98516.

[ref10] Burmistrz P.; Czepirski L.; Gazda-Grzywacz M. Carbon Dioxide Emission in Hydrogen Production Technology from Coke Oven Gas with Life Cycle Approach. E3S Web Conf. 2016, 10, 00023 10.1051/e3sconf/20161000023.

[ref11] World Steel Association. World Steel in Figures; Brussels, 2021.

[ref12] Ramírez-Santos Á. A.; Castel C.; Favre E. A Review of Gas Separation Technologies within Emission Reduction Programs in the Iron and Steel Sector: Current Application and Development Perspectives. Sep. Purif. Technol. 2018, 194, 425–442. 10.1016/j.seppur.2017.11.063.

[ref13] Yáñez M.; Ortiz A.; Brunaud B.; Grossmann I. E.; Ortiz I. Contribution of Upcycling Surplus Hydrogen to Design a Sustainable Supply Chain: The Case Study of Northern Spain. Appl. Energy 2018, 231, 777–787. 10.1016/j.apenergy.2018.09.047.

[ref14] Maestre V. M.; Ortiz A.; Ortiz I. The Role of Hydrogen-Based Power Systems in the Energy Transition of the Residential Sector. J. Chem. Technol. Biotechnol. 2022, 97, 561 10.1002/jctb.6938.

[ref15] Ortiz-Imedio R.; Caglayan D. G.; Ortiz A.; Heinrichs H.; Robinius M.; Stolten D.; Ortiz I. Power-to-Ships: Future Electricity and Hydrogen Demands for Shipping on the Atlantic Coast of Europe in 2050. Energy 2021, 228, 120660 10.1016/j.energy.2021.120660.

[ref16] Fuel Cells and Hydrogen Joint Undertaking (FCH). Hydrogen Roadmap Europe; 2019; 10.2843/249013.

[ref17] Maestre V. M.; Ortiz A.; Ortiz I. Challenges and Prospects of Renewable Hydrogen-Based Strategies for Full Decarbonization of Stationary Power Applications. Renew. Sustain. Energy Rev. 2021, 152, 111628 10.1016/j.rser.2021.111628.

[ref18] European Parliament and Council of the European Union. Hydrogen. https://ec.europa.eu/energy/topics/energy-system-integration/hydrogen_en (accessed 2022-02-15).

[ref19] International Energy Agency. Hydrogen. https://www.iea.org/reports/hydrogen (accessed 2021-10-16).

[ref20] Government of Canada. The Hydrogen Strategy. https://www.nrcan.gc.ca/climate-change/canadas-green-future/the-hydrogen-strategy/23080 (accessed 2021-11-07).

[ref21] Hydrogen Council. Hydrogen Insights Report 2021; 2021.

[ref22] Bloomberg New Energy Finance. Hydrogen Economy Outlook; Singapore, 2020.

[ref23] Zhang W.; Xie H.; Yu Z.; Wang P.; Wang Z.; Yu Q. Steam Reforming of Tar from Raw Coke Oven Gas over Bifunctional Catalysts: Reforming Performance for H2 Production. Environ. Prog. Sustain. Energy 2021, 40 (2), 1–11. 10.1002/ep.13501.

[ref24] de Oliveira Carneiro L.; de Vasconcelos S. F.; de Farias Neto G. W.; Brito R. P.; Brito K. D. Improving H2S Removal in the Coke Oven Gas Purification Process. Sep. Purif. Technol. 2021, 257, 117862 10.1016/j.seppur.2020.117862.

[ref25] Kohl A.; Nielsen R. Gas Purification, 5th ed.; Gulf Publishing: Houston, TX, 1997.

[ref26] Massey M. J.; Dunlap R. W. Economics and Alternatives for Sulfur Removal from Coke Oven Gas. J. Air Pollut. Control Assoc. 1975, 25 (10), 1019–1027. 10.1080/00022470.1975.10470173.

[ref27] Ertl G.; Knözinger H.; Weitkamp J. Handbook of Heterogeneous Catalysis; VCH Verlagsgesellschaft: Weinheim, Germany, 2008; Vol. 1–5, 10.1002/9783527610044.

[ref28] Fluenta. The Petrochemicals industry: breaking down BTX. https://www.fluenta.com/the-petrochemicals-industry-breaking-down-btx/ (accessed 2021-11-11).

[ref29] Chevron Phillips. Product Stewardship Summary Benzene, Toluene, Xylene Mixture (BTX)/Hydrotreated Pygas (HPG); 2011.

[ref30] Grosick H. A.; Kovacic J. E. Coke-Oven Gas and Effluent Treatment. In Chemistry of Coal Utilization; Elliott M. A., Ed.; John Wiley & Sons: New York, 1981; pp 1087–1151.

[ref31] Tomlinson T.; Finnb A. Hydrogen from Off-Gases. In The membrane alternative: Energy implication for industry; Howell J. A., Ed.; Elsevier Science Publishers: Essex, England, 1990; pp 79–85.

[ref32] Brunetti A.; Barbieri G.; Drioli E. Membrane Applications in Oil Refining and Petrochemical Industrial. In Handbook Of Membrane Separations: Chemical,Pharmaceutical, Food and Biotechnological Applications; Pabby A. K., Rizvi S. S. H., Sastre A. M., Eds.; CRC Press: Boca Raton, FL, 2015; pp 77–100.

[ref33] Elsherif M.; Manan Z. A.; Kamsah M. Z. State-of-the-Art of Hydrogen Management in Refinery and Industrial Process Plants. J. Nat. Gas Sci. Eng. 2015, 24, 346–356. 10.1016/j.jngse.2015.03.046.

[ref34] Wiessner F. G. Basics and Industrial Applications of Pressure Swing Adsorption (PSA), the Modern Way to Separate Gas. Gas Sep. Purif. 1988, 2 (3), 115–119. 10.1016/0950-4214(88)80026-4.

[ref35] Sircar S.; C. Golden T. Pressure Swing Adsorption Technology for Hydrogen Production. In Hydrogen and Syngas Production and Purification Technologies; Liu K., Song C., Subramani V., Eds.; AIChE: New York, USA, 2010; pp 414–450, 10.1002/9780470561256.ch10.

[ref36] Yáñez M.; Relvas F.; Ortiz A.; Gorri D.; Mendes A.; Ortiz I. PSA Purification of Waste Hydrogen from Ammonia Plants to Fuel Cell Grade. Sep. Purif. Technol. 2020, 240, 116334 10.1016/j.seppur.2019.116334.

[ref37] LeVan M. D.; Carta G.; Yon C. M. Adsorption and Ion Exchange. In Perry’s Chemical Engineers Handbook; Perry R. H., Green D. W., Maloney J. O., Eds.; McGraw-Hill: New York, 1997.

[ref38] Shen J.; Wang Z. Z.; Yang H. W.; Yao R. S. A New Technology for Producing Hydrogen and Adjustable Ratio Syngas from Coke Ove Gas. Energy and Fuels 2007, 21 (6), 3588–3592. 10.1021/ef700217j.

[ref39] Grande C. A. PSA Technology for H2 Separation. In Hydrogen Science and Engineering: Materials, Processes, Systems and Technology; Stolten D., Bernd E., Eds.; Wiley-VCH: Weinheim, Germany, 2016; Vol. 1, pp 491–508, 10.1002/9783527674268.ch21.

[ref40] Relvas F.; Whitley R. D.; Silva C.; Mendes A. Single-Stage Pressure Swing Adsorption for Producing Fuel Cell Grade Hydrogen. Ind. Eng. Chem. Res. 2018, 57 (14), 5106–5118. 10.1021/acs.iecr.7b05410.

[ref41] Chen X.; Hu F.; Lian Z.; Zhao Y. Coke Oven Gas Hydrogen Generation Process. Patent. CN103407963A, 2013.

[ref42] Sun X.; Li K.; Qiu Y.; Liu G.; Dong X. System for Comprehensively Utilizing Hydrogen Resources in Coke Oven Gas. Patent. CN210287253U, 2019.

[ref43] Tsukuda Y.; Masai S. Production of Hydrogen Gas Using Coke Oven Gas As Raw Material. Patent. JPS62153102A, 1985.

[ref44] Zeng Q.; Wang S.; Chen C. Coke Oven Gas Hydrogen Production Technology. Patent. CN107512702A, 2017.

[ref45] Air Liquide Engineering & Construction. Vacuum Swing Adsorption. engineering-airliquide.com/vacuum-swing-adsorption (accessed 2021-11-10).

[ref46] Linde AG. Oxygen Generation by Vacuum Pressure Swing Adsorption; Pullach, Germany, 2017.

[ref47] Golmakani A.; Fatemi S.; Tamnanloo J. Investigating PSA, VSA, and TSA Methods in SMR Unit of Refineries for Hydrogen Production with Fuel Cell Specification. Sep. Purif. Technol. 2017, 176, 73–91. 10.1016/j.seppur.2016.11.030.

[ref48] Delgado J. A.; Agueda V. I.; Uguina M. A.; Sotelo J. L.; Brea P. Hydrogen Recovery from Off-Gases with Nitrogen-Rich Impurity by Pressure Swing Adsorption Using CaX and 5A Zeolites. Adsorption 2015, 21 (1–2), 107–123. 10.1007/s10450-015-9654-z.

[ref49] Ahn H.; Yang J.; Lee C. H. Effects of Feed Composition of Coke Oven Gas on a Layered Bed H2 PSA Process. Adsorption 2001, 7 (4), 339–356. 10.1023/A:1013138221227.

[ref50] Jee J. G.; Kim M. B.; Lee C. H. Adsorption Characteristics of Hydrogen Mixtures in a Layered Bed: Binary, Ternary, and Five-Component Mixtures. Ind. Eng. Chem. Res. 2001, 40 (3), 868–878. 10.1021/ie0005046.

[ref51] Lopes F. V. S.; Grande C. A.; Rodrigues A. E. Fast-Cycling VPSA for Hydrogen Purification. Fuel 2012, 93, 510–523. 10.1016/j.fuel.2011.07.005.

[ref52] Yang J.; Lee C. H.; Chang J. W. Separation of Hydrogen Mixtures by a Two-Bed Pressure Swing Adsorption Process Using Zeolite 5A. Ind. Eng. Chem. Res. 1997, 36 (7), 2789–2798. 10.1021/ie960728h.

[ref53] Li H.; Liao Z.; Sun J.; Jiang B.; Wang J.; Yang Y. Modelling and Simulation of Two-Bed PSA Process for Separating H2 from Methane Steam Reforming. Chinese J. Chem. Eng. 2019, 27 (8), 1870–1878. 10.1016/j.cjche.2018.11.022.

[ref54] Cho K.; Kim J.; Park J. H.; Jung T.; Beum H. T.; Cho D. W.; Rhee Y. W.; Han S. S. High CO Adsorption Capacity, and CO Selectivity to CO2, N2, H2, and CH4 of CuCl/Bayerite Adsorbent. Microporous Mesoporous Mater. 2019, 277, 142–148. 10.1016/j.micromeso.2018.10.010.

[ref55] Kwon S.; You Y.; Lim H.; Lee J.; Chang T. S.; Kim Y.; Lee H.; Kim B. S. Selective CO Adsorption Using Sulfur-Doped Ni Supported by Petroleum-Based Activated Carbon. J. Ind. Eng. Chem. 2020, 83, 289–296. 10.1016/j.jiec.2019.11.041.

[ref56] Wu Y.; Chen Z.; Li B.; Xing J.; Liu H.; Tong Y.; Tian P.; Xu Y.; Liu Z. Highly Selective Adsorption of CO over N2 on CuCl-Loaded SAPO-34 Adsorbent. J. Energy Chem. 2019, 36, 122–128. 10.1016/j.jechem.2019.07.013.

[ref57] European Parliament and Council of the European Union. Directive 2014/94/EU of the European Parliament and of the Council of 22 October 2014 on the Deployment of Alternative Fuels Infrastructure. 2014.

[ref58] Kluiters S. C. A. Status Review on Membrane Systems for Hydrogen Separation; Petten, The Netherlands, 2004.

[ref59] Adhikari S.; Fernando S. Hydrogen Membrane Separation Techniques. Ind. Eng. Chem. Res. 2006, 45 (3), 875–881. 10.1021/ie050644l.

[ref60] Baker R.; Wijmans J. The Solution-Diffusion Model: A Review. J. Membr. Sci. 1995, 107, 1–21. 10.1016/0376-7388(95)00102-I.

[ref61] Perry J. D.; Nagai K.; Koros W. J. Polymer Membranes for Hydrogen Separations. MRS Bull. 2006, 31 (10), 745–749. 10.1557/mrs2006.187.

[ref62] Brinkmann T.; Shishatskiy S. Hydrogen Separation with Polymeric Membranes. In Hydrogen Science and Engineering: Materials, Processes, Systems and Technology; Stolten D., Bernd E., Eds.; Wiley-VCH: Weinheim, Germany, 2016; Vol. 1, pp 509–541. 10.1002/9783527674268.ch22.

[ref63] Air Products. PRISM® Membrane Systems for Petrochemical Applications; Pennsylvania, USA, 2015.

[ref64] Air Liquide Engineering & Construction. Hydrogen Membrane Overview. Advanced Membrane Technology for Hydrogen Purification and Recovery; Newport, USA, 2016.

[ref65] Generon. Hydrogen Recovery GENERON Membrane Technology; Houston, Texas, 2016.

[ref66] Evonik. Sepuran Noble. Membrane Technology for Efficient Hydrogen Generation; Schörfling, Austria, 2019.

[ref67] Honeywell. UOP Polysep Membrane Systems for Hydrogen Recovery and Purification; Des Plaines, Illinois, 2016.

[ref68] Basile A.; Mozia S.; Molinari R. Current Trends and Future Developments on (Bio-) Membranes: Photocatalytic Membranes and Photocatalytic Membrane Reactors; Elsevier: Amsterdam, 2018; 10.1016/C2016-0-02120-4.

[ref69] H2site. Soluciones para producción in-situ de hidrógeno de alta calidad. https://www.h2site.eu/es/ (accessed 2022-01-20).

[ref70] Shirasaki Y.; Yasuda I. Membrane Reactor for Hydrogen Production from Natural Gas at the Tokyo Gas Company: A Case Study. In Handbook of Membrane Reactors; Basile A., Ed.; Woodhead Publishing Limited: Oxford, 2013; 10.1533/9780857097347.2.487.

[ref71] Vente J. F.; Exter M. J. D.; Delft Y. C. Van; Groot A. D. Hydrogen Separation Pd Based Alloy Membranes: Recent Developments and Challenges; Amersfoort, 2009.

[ref72] Peters T. A.; Rørvik P. M.; Sunde T. O.; Stange M.; Roness F.; Reinertsen T. R.; Ræder J. H.; Larring Y.; Bredesen R. Palladium (Pd) Membranes as Key Enabling Technology for Pre-Combustion CO2 Capture and Hydrogen Production. Energy Procedia 2017, 114 (1876), 37–45. 10.1016/j.egypro.2017.03.1144.

[ref73] Shen J. Y.; Jun Y. S. Technique for Preparing Synthesis Gas from Coke Oven Gas. Patent. CN1872663, 2006.

[ref74] Songbo W.; Zaoshleng L.; Zhixue L.; Guanghui W.; Hongbing C.; Junbo S.; Xiangyong L.; Sumei C. Method for Extracting Hydrogen Gas with Purity from Coke Oven Gas by Metal Palladium Membrane Separation Technique. Patent. CN101648105, 2010.

[ref75] Xu H.; Meng F.; Dong E.; Li A. Method for Extracting High Purity Hydrogen from Coke Oven Gas Reformed Gas. Patent. CN104176706, 2014.

[ref76] Pacheco Tanaka D. A.; Medrano J. A.; Viviente Sole J. L.; Gallucci F. 1-Metallic Membranes for Hydrogen Separation. In Current Trends and Future Developments on (Bio-) Membranes; Basile A., Ghasemzadeh K., Eds.; Elsevier Inc.: 2020; pp 1–29, 10.1016/B978-0-12-818332-8.00001-6.

[ref77] Abdollahi M.; Yu J.; Liu P. K. T.; Ciora R.; Sahimi M.; Tsotsis T. T. Ultra-Pure Hydrogen Production from Reformate Mixtures Using a Palladium Membrane Reactor System. J. Membr. Sci. 2012, 390–391, 32–42. 10.1016/j.memsci.2011.10.053.

[ref78] Conde J. J.; Maroño M.; Sánchez-Hervás J. M. Pd-Based Membranes for Hydrogen Separation: Review of Alloying Elements and Their Influence on Membrane Properties. Sep. Purif. Rev. 2017, 46 (2), 152–177. 10.1080/15422119.2016.1212379.

[ref79] Gallucci F.; Fernandez E.; Corengia P.; van Sint Annaland M. Recent Advances on Membranes and Membrane Reactors for Hydrogen Production. Chem. Eng. Sci. 2013, 92, 40–66. 10.1016/j.ces.2013.01.008.

[ref80] Al-Mufachi N. A.; Rees N. V.; Steinberger-Wilkens R. Hydrogen Selective Membranes: A Review of Palladium-Based Dense Metal Membranes. Renew. Sustain. Energy Rev. 2015, 47, 540–551. 10.1016/j.rser.2015.03.026.

[ref81] Itoh N.; Akiha T.; Sato T. Preparation of Thin Palladium Composite Membrane Tube by a CVD Technique and Its Hydrogen Permselectivity. Catal. Today 2005, 104, 231–237. 10.1016/j.cattod.2005.03.048.

[ref82] Pereira A. I.; Pérez P.; Rodrigues S. C.; Mendes A.; Madeira L. M.; Tavares C. J. Deposition of Pd-Ag Thin Film Membranes on Ceramic Supports for Hydrogen Purification/Separation. Mater. Res. Bull. 2015, 61, 528–533. 10.1016/j.materresbull.2014.10.055.

[ref83] Shi L.; Goldbach A.; Zeng G.; Xu H. Preparation and Performance of Thin-Layered PdAu/Ceramic Composite Membranes. Int. J. Hydrogen Energy 2010, 35, 4201–4208. 10.1016/j.ijhydene.2010.02.048.

[ref84] Ding Y. Perspective on Gas Separation Membrane Materials from Process Economics Point of View. Ind. Eng. Chem. Res. 2020, 59 (2), 556–568. 10.1021/acs.iecr.9b05975.

[ref85] Pathare R.; Agrawal R. Design of Membrane Cascades for Gas Separation. J. Membr. Sci. 2010, 364 (1–2), 263–277. 10.1016/j.memsci.2010.08.029.

[ref86] Zarca R.; Ortiz A.; Gorri D.; Biegler L. T.; Ortiz I. Optimization of Multistage Olefin/Paraffin Membrane Separation Processes through Rigorous Modeling. AIChE J. 2019, 65 (6), e16588 10.1002/aic.16588.

[ref87] Baker R. W. Membrane Technology and Applications, 3rd ed.; John Wiley & Sons: Chichester, 2012; 10.1002/9781118359686.

[ref88] Yáñez M.; Ortiz A.; Gorri D.; Ortiz I. Comparative Performance of Commercial Polymeric Membranes in the Recovery of Industrial Hydrogen Waste Gas Streams. Int. J. Hydrogen Energy 2021, 46 (33), 17507–17521. 10.1016/j.ijhydene.2020.04.026.

[ref89] Ansaloni L.; Louradour E.; Radmanesh F.; van Veen H.; Pilz M.; Simon C.; Benes N. E.; Peters T. A. Upscaling PolyPOSS-Imide Membranes for High Temperature H2 Upgrading. J. Membr. Sci. 2021, 620, 118875 10.1016/j.memsci.2020.118875.

[ref90] Radmanesh F.; Pilz M.; Ansaloni L.; Peters T. A.; Louradour E.; van Veen H.; Høvik D.; Hempenius M. A.; Benes N. E. Comparing Amine- and Ammonium Functionalized Silsesquioxanes for Large Scale Synthesis of Hybrid Polyimide High-Temperature Gas Separation Membranes. J. Membr. Sci. 2021, 637, 119524 10.1016/j.memsci.2021.119524.

[ref91] Bernardo G.; Araújo T.; da Silva Lopes T.; Sousa J.; Mendes A. Recent Advances in Membrane Technologies for Hydrogen Purification. Int. J. Hydrogen Energy 2020, 45 (12), 7313–7338. 10.1016/j.ijhydene.2019.06.162.

[ref92] Acharya N. K.; Kulshrestha V.; Awasthi K.; Jain A. K.; Singh M.; Vijay Y. K. Hydrogen Separation in Doped and Blend Polymer Membranes. Int. J. Hydrogen Energy 2008, 33 (1), 327–331. 10.1016/j.ijhydene.2007.07.030.

[ref93] Kapantaidakis G. G.; Kaldis S. P.; Dabou X. S.; Sakellaropoulos G. P. Gas Permeation through PSF-PI Miscible Blend Membranes. J. Membr. Sci. 1996, 110 (2), 239–247. 10.1016/0376-7388(95)00265-0.

[ref94] Hosseini S. S.; Chung T. S. Carbon Membranes from Blends of PBI and Polyimides for N2/CH4 and CO2/CH4 Separation and Hydrogen Purification. J. Membr. Sci. 2009, 328 (1–2), 174–185. 10.1016/j.memsci.2008.12.005.

[ref95] Bos A.; Pünt I.; Strathmann H.; Wessling M. Suppression of Gas Separation Membrane Plasticization by Homogeneous Polymer Blending. AIChE J. 2001, 47 (5), 1088–1093. 10.1002/aic.690470515.

[ref96] Hosseini S.; Peng N.; Chung T. Gas Separation Membranes Developed through Integration of Polymer Blending and Dual-Layer Hollow Fiber Spinning Process for Hydrogen and Natural Gas Enrichments. J. Membr. Sci. 2010, 349, 156–166. 10.1016/j.memsci.2009.11.043.

[ref97] Lei L.; Pan F.; Lindbråthen A.; Zhang X.; Hillestad M.; Nie Y.; Bai L.; He X.; Guiver M. D. Carbon Hollow Fiber Membranes for a Molecular Sieve with Precise-Cutoff Ultramicropores for Superior Hydrogen Separation. Nat. Commun. 2021, 12, 268 10.1038/s41467-020-20628-9. 33431865 PMC7801458

[ref98] Xu R.; He L.; Li L.; Hou M.; Wang Y.; Zhang B.; Liang C.; Wang T. Ultraselective Carbon Molecular Sieve Membrane for Hydrogen Purification. J. Energy Chem. 2020, 50, 16–24. 10.1016/j.jechem.2020.03.008.

[ref99] Chuah C. Y.; Lee J.; Bae T. H. Graphene-Based Membranes for H2 Separation: Recent Progress and Future Perspective. Membranes (Basel) 2020, 10, 336 10.3390/membranes10110336. PMC7697601 33198281

[ref100] Li H.; Song Z.; Zhang X.; Huang Y.; Li S.; Mao Y.; Ploehn H. J.; Bao Y.; Yu M. Ultrathin, Molecular-Sieving Graphene Oxide Membranes for Selective Hydrogen Separation. Science 2013, 342 (6154), 95–98. 10.1126/science.1236686. 24092739

[ref101] Huang K.; Yuan J.; Shen G.; Liu G. Graphene Oxide Membranes Supported on the Ceramic Hollow Fiber for Efficient H2 Recovery. Chinese J. Chem. Eng. 2017, 25, 752 10.1016/j.cjche.2016.11.010.

[ref102] Yoo B. M.; Shin J. E.; Lee H. D.; Park H. B. Graphene and Graphene Oxide Membranes for Gas Separation Applications. Curr. Opin. Chem. Eng. 2017, 16, 39–47. 10.1016/j.coche.2017.04.004.

[ref103] Huang G.; Ghalei B.; Isfahani A. P.; Karahan H. E.; Terada D.; Li C.; Tsujimoto M.; Yamaguchi D.; Sugimoto K.; Igarashi R.; Chang B. K.; Li T.; Shirakawa M.; Sivaniah E. Overcoming Humidity-Induced Swelling of Graphene Oxide-Based Hydrogen Membranes Using Charge-Compensating Nanodiamonds. Nat. Energy 2021, 6, 1176–1187. 10.1038/s41560-021-00946-y.

[ref104] Chuah C. Y.; Jiang X.; Goh K.; Wang R. Recent Progress in Mixed-Matrix Membranes for Hydrogen Separation. Membranes (Basel) 2021, 11, 666 10.3390/membranes11090666. 34564483 PMC8466440

[ref105] Ünügül T.; Nigiz F. U. Hydrogen Purification Using Natural Zeolite-Loaded Hydroxyethyl Cellulose Membrane. Int. J. Energy Res. 2022, 46, 1826–1836. 10.1002/er.7299.

[ref106] Rezakazemi M.; Shahidi K.; Mohammadi T. Sorption Properties of Hydrogen-Selective PDMS/Zeolite 4A Mixed Matrix Membrane. Int. J. Hydrogen Energy 2012, 37 (22), 17275–17284. 10.1016/j.ijhydene.2012.08.109.

[ref107] Peydayesh M.; Mohammadi T.; Bakhtiari O. Effective Hydrogen Purification from Methane via Polyimide Matrimid® 5218- Deca-Dodecasil 3R Type Zeolite Mixed Matrix Membrane. Energy 2017, 141, 2100–2107. 10.1016/j.energy.2017.11.101.

[ref108] Ahmad J.; Hägg M. B. Preparation and Characterization of Polyvinyl Acetate/Zeolite 4A Mixed Matrix Membrane for Gas Separation. J. Membr. Sci. 2013, 427, 73–84. 10.1016/j.memsci.2012.09.036.

[ref109] Khan A. L.; Cano-Odena A.; Gutiérrez B.; Minguillón C.; Vankelecom I. F. J. Hydrogen Separation and Purification Using Polysulfone Acrylate-Zeolite Mixed Matrix Membranes. J. Membr. Sci. 2010, 350 (1–2), 340–346. 10.1016/j.memsci.2010.01.009.

[ref110] Fernández-Castro P.; Ortiz A.; Gorri D. Exploring the Potential Application of Matrimid® and ZIFs-Based Membranes for Hydrogen Recovery: A Review. Polymers (Basel) 2021, 13 (8), 1292 10.3390/polym13081292. 33921024 PMC8071404

[ref111] Diestel L.; Wang N.; Schulz A.; Steinbach F.; Caro J. Matrimid-Based Mixed Matrix Membranes: Interpretation and Correlation of Experimental Findings for Zeolitic Imidazolate Frameworks as Fillers in H2/CO2 Separation. Ind. Eng. Chem. Res. 2015, 54 (3), 1103–1112. 10.1021/ie504096j.

[ref112] Carter D.; Tezel F. H.; Kruczek B.; Kalipcilar H. Investigation and Comparison of Mixed Matrix Membranes Composed of Polyimide Matrimid with ZIF-8, Silicalite, and SAPO-34. J. Membr. Sci. 2017, 544, 35–46. 10.1016/j.memsci.2017.08.068.

[ref113] Song Q.; Nataraj S. K.; Roussenova M. V.; Tan J. C.; Hughes D. J.; Li W.; Bourgoin P.; Alam M. A.; Cheetham A. K.; Al-Muhtaseb S. A.; Sivaniah E. Zeolitic Imidazolate Framework (ZIF-8) Based Polymer Nanocomposite Membranes for Gas Separation. Energy Environ. Sci. 2012, 5 (8), 8359–8369. 10.1039/c2ee21996d.

[ref114] Li B.; He G.; Jiang X.; Dai Y.; Ruan X. Pressure Swing Adsorption/Membrane Hybrid Processes for Hydrogen Purification with a High Recovery. Front. Chem. Sci. Eng. 2016, 10 (2), 255–264. 10.1007/s11705-016-1567-1.

[ref115] Lin L.; Tian Y.; Su W.; Luo Y.; Chen C.; Jiang L. Techno-Economic Analysis and Comprehensive Optimization of an on-Site Hydrogen Refuelling Station System Using Ammonia: Hybrid Hydrogen Purification with Both High H2 purity and High Recovery. Sustain. Energy Fuels 2020, 4 (6), 3006–3017. 10.1039/C9SE01111K.

[ref116] Hashim S. S.; Somalu M. R.; Loh K. S.; Liu S.; Zhou W.; Sunarso J. Perovskite-Based Proton Conducting Membranes for Hydrogen Separation: A Review. Int. J. Hydrogen Energy 2018, 43 (32), 15281–15305. 10.1016/j.ijhydene.2018.06.045.

[ref117] Phair J. W.; Badwal S. P. S. Review of Proton Conductors for Hydrogen Separation. Ionics (Kiel) 2006, 12 (2), 103–115. 10.1007/s11581-006-0016-4.

[ref118] Tao Z.; Yan L.; Qiao J.; Wang B.; Zhang L.; Zhang J. A Review of Advanced Proton-Conducting Materials for Hydrogen Separation. Prog. Mater. Sci. 2015, 74, 1–50. 10.1016/j.pmatsci.2015.04.002.

[ref119] Indarto A.; Palguandi J. Syngas. Production, Applications and Environmetal Impact; Nova Science: New York, 2013.

[ref120] U.S. Department of Energy Office of Fossil Energy and Carbon Management. Hydrogen Production: Natural Gas Reforming. https://www.energy.gov/eere/fuelcells/hydrogen-production-natural-gas-reforming (accessed 2021-11-05).

[ref121] Van Beurden P. On The Catalytic Aspects Of Steam Reforming Methane - A Literature Survey; Petten, The Netherlands, 2004.

[ref122] García L. Hydrogen Production by Steam Reforming of Natural Gas and Other Nonrenewable Feedstocks. In Compendium of Hydrogen Energy; Subramani V., Basile A., Veziroğlu T. N., Eds.; Elsevier Ltd.: Amsterdam, 2015; pp 83–107, 10.1016/b978-1-78242-361-4.00004-2.

[ref123] Zhang H.; Sun Z.; Hu Y. H. Steam Reforming of Methane: Current States of Catalyst Design and Process Upgrading. Renew. Sustain. Energy Rev. 2021, 149, 111330 10.1016/j.rser.2021.111330.

[ref124] Ma B.; Deng C.; Chen H.; Zhu M.; Yang M.; Feng X. Hybrid Separation Process of Refinery Off-Gas toward Near-Zero Hydrogen Emission: Conceptual Design and Techno-Economic Analysis. Ind. Eng. Chem. Res. 2020, 59 (18), 8715–8727. 10.1021/acs.iecr.0c00143.

[ref125] Amiri T. Y.; Ghasemzageh K.; Iulianelli A. Membrane Reactors for Sustainable Hydrogen Production through Steam Reforming of Hydrocarbons: A Review. Chem. Eng. Process. - Process Intensif. 2020, 157, 108148 10.1016/j.cep.2020.108148.

[ref126] Membrane Reactors for Hydrogen Production Processes; De Falco M., Marrelli L., Ianquaniello G., Eds.; Springer-Verlag: London, 2011; 10.1007/978-0-85729-151-6.

[ref127] De Falco M.; Barba D.; Cosenza S.; Iaquaniello G.; Farace A.; Giacobbe F. G. Reformer and Membrane Modules Plant to Optimize Natural Gas Conversion to Hydrogen. Asia-Pacific J. Chem. Eng. 2009, 4, 259–269. 10.1002/apj.241.

[ref128] Murmura M. A.; Cerbelli S.; Annesini M. C. Modeling Fixed Bed Membrane Reactors for Hydrogen Production through Steam Reforming Reactions: A Critical Analysis. Membranes (Basel) 2018, 8 (2), 34 10.3390/membranes8020034. PMC6026897 29921794

[ref129] Chen Y.; Zhang J.; Qin Y.; Zhao Y.; Longji Y. Steam Reforming Device and Method for Producing Hydrogen and Fixing Carbon through Coke Oven Gas in Synergic Mode. Patent. CN105197887A, 2015.

[ref130] Fusheng Y.; Yung L.; Yuqi Y.; Shiwei Z.; Fa Z.; Lingxiao W. Method for Preparing Hydrogen with Different Purity Levels by Use of Semi-Coke Oven as and System Thereof. Patent. CN103359688A, 2013.

[ref131] Chen Y.; Peng R.; Xiao Y.; Zhang B.; Yan W.; Zhao Y.; Zhang J. Efficient Hydrogen Production from Coke Oven Gas by Sorption-Enhanced Steam Reforming in a Membrane-Assisted Fluidized Bed Reactor. Energy and Fuels 2019, 33 (11), 11420–11438. 10.1021/acs.energyfuels.9b02548.

[ref132] Chen Y.; Zhang B.; Peng R.; Chuai X.; Cui X.; Kang B.; Yan W.; Zhang J. Comprehensive Modeling of Sorption-Enhanced Steam Reforming of Coke Oven Gas in a Fluidized Bed Membrane Reactor. Energy and Fuels 2020, 34, 3065–3086. 10.1021/acs.energyfuels.9b04433.

[ref133] Wu R.; Wu S. F. The ReSER-COG Process for Hydrogen Production on a Ni-CaO/Al 2O3 Complex Catalyst. Int. J. Hydrogen Energy 2013, 38 (27), 11818–11825. 10.1016/j.ijhydene.2013.06.117.

[ref134] Malerød-Fjeld H.; Clark D.; Yuste-Tirados I.; Zanón R.; Catalán-Martinez D.; Beeaff D.; Morejudo S. H.; Vestre P. K.; Norby T.; Haugsrud R.; Serra J. M.; Kjølseth C. Thermo-Electrochemical Production of Compressed Hydrogen from Methane with near-Zero Energy Loss. Nat. Energy 2017, 2 (12), 923–931. 10.1038/s41560-017-0029-4.

[ref135] Li G.; Cheng H.; Zhao H.; Lu X.; Xu Q.; Wu C. Hydrogen Production by CO2 Reforming of CH4 in Coke Oven Gas over Ni–Co/MgAl2O4 Catalysts. Catal. Today 2018, 318, 46–51. 10.1016/j.cattod.2017.12.033.

[ref136] Angeli S. D.; Gossler S.; Lichtenberg S.; Kass G.; Agrawal A. K.; Valerius M.; Kinzel K. P.; Deutschmann O. Reduction of CO2 Emission from Off-Gases of Steel Industry by Dry Reforming of Methane. Angew. Chemie - Int. Ed. 2021, 60 (21), 11852–11857. 10.1002/anie.202100577. PMC8251717 33661578

[ref137] Kim A. R.; Lee H. Y.; Cho J. M.; Choi J. H.; Bae J. W. Ni/M-Al2O3 (M = Sm, Ce or Mg) for Combined Steam and CO2 Reforming of CH4 from Coke Oven Gas. J. CO2 Util. 2017, 21, 211–218. 10.1016/j.jcou.2017.07.011.

[ref138] Zhang Y.; Jiao L.; Yong L.; Weizhong D.; Xionggang L. Perovskite-Type Oxygen-Permeable Membrane BaCo0.7F 0.2Nb0.1O3-δ for Partial Oxidation of Methane in Coke Oven Gas to Hydrogen. Rare Met. 2010, 29 (3), 231–237. 10.1007/s12598-010-0040-4.

[ref139] Cheng H.; Yao W.; Lu X.; Zhou Z.; Li C.; Liu J. Structural Stability and Oxygen Permeability of BaCo0.7Fe0.2M0.1O3-δ (M = Ta, Nb, Zr) Ceramic Membranes for Producing Hydrogen from Coke Oven Gas. Fuel Process. Technol. 2015, 131, 36–44. 10.1016/j.fuproc.2014.11.004.

[ref140] Zhang Y.; Li Q.; Shen P.; Liu Y.; Yang Z.; Ding W.; Lu X. Hydrogen Amplification of Coke Oven Gas by Reforming of Methane in a Ceramic Membrane Reactor. Int. J. Hydrogen Energy 2008, 33 (13), 3311–3319. 10.1016/j.ijhydene.2008.04.015.

[ref141] Zhang Y.; Liu J.; Ding W.; Lu X. Performance of an Oxygen-Permeable Membrane Reactor for Partial Oxidation of Methane in Coke Oven Gas to Syngas. Fuel 2011, 90 (1), 324–330. 10.1016/j.fuel.2010.08.027.

[ref142] Yao W.; Cheng H.; Wang P.; Lu X.; Zou X.; Xu Q. Hydrogen Production by Catalytic Partial Oxidation of Coke Oven Gas in BaCo0.7Fe0.3-XZrxO3-δ Ceramic Membrane Reactors. MATEC Web Conf. 2016, 67, 04002 10.1051/matecconf/20166704002.

[ref143] Yang Z.; Ding W.; Zhang Y.; Lu X.; Zhang Y.; Shen P. Catalytic Partial Oxidation of Coke Oven Gas to Syngas in an Oxygen Permeation Membrane Reactor Combined with NiO/MgO Catalyst. Renew. Energy 2010, 35 (12), 6239–6247. 10.1016/j.ijhydene.2009.07.103.

[ref144] Specchia S.; Fornasiero P.; Vella L.; Montini T. Syngas Production by Short Contact Time Catalytic Partial Oxidation of Methane; Nova Science: New York, 2011.

[ref145] Cheng H.; Zhang Y.; Ding W.; Lu X.; Jun Y. Oxygen-Permeating Film Reactor Used for Coke Oven Crude Gas Mixing Reforming Preparation Technique. Patent. CN101264860A, 2008.

[ref146] Ito N.; Donomae H.; Suzuki K.; Nakao K.; Isohara T. Hydrogen Gas Production Apparatus and Method. Patent. JP2016037411A, 2017.

[ref147] Yun X.; Wen Y.; Liu L.; Feng L.; Tan J.; Cao Z.; Wu D. System for Catalytic Reforming High Temperature Coke Oven Gas Prepares Still Crude Gas. Patent. CN207738447U, 2018.

[ref148] Qin Z.; Zhao Y.; Yi Q.; Shi L.; Li C.; Yan X.; Ren J.; Miao M.; Xie K. Methanation of Coke Oven Gas over Ni-Ce/γ-Al2O3 Catalyst Using a Tubular Heat Exchange Reactor: Pilot-Scale Test and Process Optimization. Energy Convers. Manag. 2020, 204, 112302 10.1016/j.enconman.2019.112302.

[ref149] Stangeland K.; Kalai D.; Li H.; Yu Z. CO2 Methanation: The Effect of Catalysts and Reaction Conditions. Energy Procedia 2017, 105 (1876), 2022–2027. 10.1016/j.egypro.2017.03.577.

[ref150] Chen Z.; Sun X. Novel Coke Oven Gas Methanation System Natural Gas Complete Equipment. Patent. CN205838937U, 2016.

[ref151] Liu Y.; Wang Y.; Huang T.; Lin L.; Yan H.; Hou Q. Methanation Natural Gas Synthesis Method through Supplying Hydrogen to Low Hydrogen-Carbon Ratio Coke Oven Gas. Patent. CN108219880A, 2018.

[ref152] Lu H.; Yang X.; Gao G.; Wang K.; Shi Q.; Wang J.; Han C.; Liu J.; Tong M.; Liang X.; Li C. Mesoporous Zirconia-Modified Clays Supported Nickel Catalysts for CO and CO2 Methanation. Int. J. Hydrogen Energy 2014, 39 (33), 18894–18907. 10.1016/j.ijhydene.2014.09.076.

[ref153] Qin Z.; Ren J.; Miao M.; Li Z.; Lin J.; Xie K. The Catalytic Methanation of Coke Oven Gas over Ni-Ce/Al2O3 Catalysts Prepared by Microwave Heating: Effect of Amorphous NiO Formation. Appl. Catal. B Environ. 2015, 164, 18–30. 10.1016/j.apcatb.2014.08.047.

[ref154] Moulijn J. A.; Mankkee M.; Van Diepen A. E. Production of Synthesis Gas. In Chemical Process Technology; John Wiley & Sons: West Sussex, UK, 2013; pp 166–167.

[ref155] Zhang L.; Xie W.; Ren Z. Combustion Stability Analysis for Non-Standard Low-Calorific Gases: Blast Furnace Gas and Coke Oven Gas. Fuel 2020, 278, 118216 10.1016/j.fuel.2020.118216.

[ref156] Whiting R. Review of Net Calorific Values for Non-Standard Gaseous Fuels: Task 9 of the 2010 UK/DA GHG Inventory Improvement Programme; No. AEAT/ENV/R/4000; 2011.

[ref157] García García S.; Rodríguez V.; Morán H.; Mones A. A Mixed Integer Linear Programming Model for the Optimization of Steel Waste Gases in Cogeneration: A Combined Coke Oven and Converter Gas Case Study. Energies 2020, 13 (15), 3781 10.3390/en13153781.

[ref158] Zhao H.; Jiang T.; Hou H. Performance Analysis of the SOFC-CCHP System Based on H2O/Li-Br Absorption Refrigeration Cycle Fueled by Coke Oven Gas. Energy 2015, 91, 983–993. 10.1016/j.energy.2015.08.087.

[ref159] Goldmeer J. Is there a Hydrogen Future for Your Gas Turbine? https://www.esig.energy/is-there-a-hydrogen-future-for-your-gas-turbine/ (accessed 2021-11-19).

[ref160] Mom A. J. A. Introduction to Gas Turbines. In Modern Gas Turbine Systems; Jansohn P., Ed.; Woodhead Publishing Limited: Sawston, 2013; 10.1533/9780857096067.1.3.

[ref161] Mcmillan R.; Marriott D. Fuel-Flexible Gas Turbine Cogeneration; Siemens: Kuala Lumpur, Malaysia, 2008.

[ref162] Shi W.; An L.; Chen H.; Zhang X. Performance Simulation of Gas Turbine Combined Cycle with Coke Oven Gas as Fuel. Asia-Pacific Power Energy Eng. Conf. APPEEC 2009, 1 10.1109/APPEEC.2009.4918594.

[ref163] Jones R.; Goldmeer J.; Monetti B. Addressing Gas Turbine Fuel Flexibility; GER4601 (05/11) revB, 2011.

[ref164] Escalante Soberanis M. A.; Fernandez A. M. A Review on the Technical Adaptations for Internal Combustion Engines to Operate with Gas/Hydrogen Mixtures. Int. J. Hydrogen Energy 2010, 35 (21), 12134–12140. 10.1016/j.ijhydene.2009.09.070.

[ref165] Sopena C.; Diéguez P. M.; Sáinz D.; Urroz J. C.; Guelbenzu E.; Gandía L. M. Conversion of a Commercial Spark Ignition Engine to Run on Hydrogen: Performance Comparison Using Hydrogen and Gasoline. Int. J. Hydrogen Energy 2010, 35 (3), 1420–1429. 10.1016/j.ijhydene.2009.11.090.

[ref166] Verhelst S.; Verstraeten S.; Sierens R. A Comprehensive Overview of Hydrogen Engine Design Features. Proc. Inst. Mech. Eng. Part D J. Automob. Eng. 2007, 221 (8), 911–920. 10.1243/09544070JAUTO141.

[ref167] White C. M.; Steeper R. R.; Lutz A. E. The Hydrogen-Fueled Internal Combustion Engine: A Technical Review. Int. J. Hydrogen Energy 2006, 31 (10), 1292–1305. 10.1016/j.ijhydene.2005.12.001.

[ref168] Navale S. J.; Kulkarni R. R.; Thipse S. S. An Experimental Study on Performance, Emission and Combustion Parameters of Hydrogen Fueled Spark Ignition Engine with the Timed Manifold Injection System. Int. J. Hydrogen Energy 2017, 42 (12), 8299–8309. 10.1016/j.ijhydene.2017.01.059.

[ref169] Verhelst S.; Maesschalck P.; Rombaun N.; Sierens R. Efficiency Comparison between Hydrogen and Gasoline, on a Bi-Fuel Hydrogen/Gasoline Engine. Int. J. Hydrogen Energy 2009, 34 (5), 2504–2510. 10.1016/j.ijhydene.2009.01.009.

[ref170] Yan F.; Lei X.; Wang Y. Application of Hydrogen Enriched Natural Gas in Spark Ignition IC Engines: From Fundamental Fuel Properties to Engine Performances and Emissions. Renew. Sustain. Energy Rev. 2018, 82 (Part 1), 1457–1488. 10.1016/j.rser.2017.05.227.

[ref171] Mehra R. K.; Duan H.; Juknelevicius R.; Ma F. Progress in Hydrogen Enriched Compressed Natural Gas (HCNG) Internal Combustion Engines-A Comprehensive Review. Renew. Sustain. Energy Rev. 2017, 80, 1458–1498. 10.1016/j.rser.2017.05.061.

[ref172] Diéguez P. M.; Urroz J. C.; Marcelino-Sádaba S.; Pérez-Ezcurdia A.; Benito-Amurrio M.; Sáinz D.; Gandía L. M. Experimental Study of the Performance and Emission Characteristics of an Adapted Commercial Four-Cylinder Spark Ignition Engine Running on Hydrogen-Methane Mixtures. Appl. Energy 2014, 113, 1068–1076. 10.1016/j.apenergy.2013.08.063.

[ref173] Roy M. M.; Tomita E.; Kawahara N.; Harada Y.; Sakane A. Comparison of Performance and Emissions of a Supercharged Dual-Fuel Engine Fueled by Hydrogen and Hydrogen-Containing Gaseous Fuels. Int. J. Hydrogen Energy 2011, 36 (12), 7339–7352. 10.1016/j.ijhydene.2011.03.070.

[ref174] Roy M. M.; Tomita E.; Kawahara N.; Harada Y.; Sakane A. Performance and Emissions of a Supercharged Dual-Fuel Engine Fueled by Hydrogen-Rich Coke Oven Gas. Int. J. Hydrogen Energy 2009, 34 (23), 9628–9638. 10.1016/j.ijhydene.2009.09.016.

[ref175] Szwaja S. Dilution of Fresh Charge for Reducing Combustion Knock in the Internal Combustion Engine Fueled with Hydrogen Rich Gases. Int. J. Hydrogen Energy 2019, 44 (34), 19017–19025. 10.1016/j.ijhydene.2018.10.134.

[ref176] Szwaja S. Hydrogen Rich Gases Combustion in the IC Engine. J. KONES 2009, 16 (4), 447–454.

[ref177] Naeve N.; He Y.; Deng J.; Wang M. Waste Coke Oven Gas Used as a Potential Fuel for Engines. SAE 2011 World Congress & Exhibition; SAE Tech. Pap. 2011-01-0920; 2011; 10.4271/2011-01-0920.

[ref178] Feng H.; Zhang W.; Zhang J.; Wang X.; Zhang X. Availability Analysis of a Coke Oven Gas Fueled Spark Ignition Engine. Int. J. Hydrogen Energy 2018, 43 (3), 1835–1845. 10.1016/j.ijhydene.2017.11.125.

[ref179] Ortiz-Imedio R.; Ortiz A.; Urroz J. C.; Diéguez P. M.; Gorri D.; Gandía L. M.; Ortiz I. Comparative Performance of Coke Oven Gas, Hydrogen and Methane in a Spark Ignition Engine. Int. J. Hydrogen Energy 2021, 46 (33), 17572–17586. 10.1016/j.ijhydene.2019.12.165.

[ref180] Ortiz-Imedio R.; Ortiz A.; Ortiz I. Comprehensive Analysis of the Combustion of Low Carbon Fuels (Hydrogen, Methane and Coke Oven Gas) in Spark Ignition Engine through CFD Modelling. Energy Convers. Manag. 2022, 251 (10), 114918 10.1016/j.enconman.2021.114918.

[ref181] García S.; Rodríguez V.; Luiña R.; Ortega F. Evaluation of the Synergies in Cogeneration with Steel Waste Gases Based on Life Cycle Assessment: A Combined Coke Oven and Steelmaking Gas Case Study. J. Clean. Prod. 2019, 217, 576–583. 10.1016/j.jclepro.2019.01.262.

[ref182] O.E.C.D. Environmental indicators, modelling and outlooks. https://www.oecd.org/env/indicators-modelling-outlooks/ (accessed 2022-01-23).

[ref183] Zhang Y.; Tian Z.; Chen X.; Xu X. Technology-Environment-Economy Assessment of High-Quality Utilization Routes for Coke Oven Gas. Int. J. Hydrogen Energy 2022, 47 (1), 666–685. 10.1016/j.ijhydene.2021.10.011.

[ref184] Li J.; Cheng W. Comparative Life Cycle Energy Consumption, Carbon Emissions and Economic Costs of Hydrogen Production from Coke Oven Gas and Coal Gasification. Int. J. Hydrogen Energy 2020, 45 (51), 27979–27993. 10.1016/j.ijhydene.2020.07.079.

[ref185] Abejón R.; Fernández-Ríos A.; Domínguez-Ramos A.; Laso J.; Ruiz-Salmón I.; Yáñez M.; Ortiz A.; Gorri D.; Donzel N.; Jones D.; Irabien A.; Ortiz I.; Aldaco R.; Margallo M. Hydrogen Recovery from Waste Gas Streams to Feed (High-Temperature PEM) Fuel Cells: Environmental Performance under a Life-Cycle Thinking Approach. Appl. Sci. 2020, 10 (21), 7461 10.3390/app10217461.

